# Mitochondrial Regulation of the Hippocampal Firing Rate Set Point and Seizure Susceptibility

**DOI:** 10.1016/j.neuron.2019.03.045

**Published:** 2019-06-05

**Authors:** Boaz Styr, Nir Gonen, Daniel Zarhin, Antonella Ruggiero, Refaela Atsmon, Neta Gazit, Gabriella Braun, Samuel Frere, Irena Vertkin, Ilana Shapira, Michal Harel, Leore R. Heim, Maxim Katsenelson, Ohad Rechnitz, Saja Fadila, Dori Derdikman, Moran Rubinstein, Tamar Geiger, Eytan Ruppin, Inna Slutsky

**Affiliations:** 1 Department of Physiology and Pharmacology, Sackler Faculty of Medicine, Tel Aviv University, 69978 Tel Aviv, Israel; 2 Sagol School of Neuroscience, Tel Aviv University, 69978 Tel Aviv, Israel; 3 Cancer Data Science Lab (CDSL), National Cancer Institute, NIH, Bethesda, MD, USA; 4 Department of Human Genetics and Biochemistry, Sackler Faculty of Medicine, Tel Aviv University, 69978 Tel Aviv, Israel; 5 The Goldschleger Eye Research Institute, Sackler Faculty of Medicine, Tel Aviv University, 69978 Tel Aviv, Israel; 6 Department of Neuroscience, Rappaport Faculty of Medicine and Research Institute, Technion – Israel Institute of Technology, 31096 Haifa, Israel

**Keywords:** homeostasis, set point, firing rate, hippocampus, dihydroorotate dehydrogenase, mitochondria, calcium, neuronal metabolism, epilepsy, Dravet syndrome

## Abstract

Maintaining average activity within a set-point range constitutes a fundamental property of central neural circuits. However, whether and how activity set points are regulated remains unknown. Integrating genome-scale metabolic modeling and experimental study of neuronal homeostasis, we identified mitochondrial dihydroorotate dehydrogenase (DHODH) as a regulator of activity set points in hippocampal networks. The DHODH inhibitor teriflunomide stably suppressed mean firing rates via synaptic and intrinsic excitability mechanisms by modulating mitochondrial Ca^2+^ buffering and spare respiratory capacity. Bi-directional activity perturbations under DHODH blockade triggered firing rate compensation, while stabilizing firing to the lower level, indicating a change in the firing rate set point. *In vivo*, teriflunomide decreased CA3-CA1 synaptic transmission and CA1 mean firing rate and attenuated susceptibility to seizures, even in the intractable Dravet syndrome epilepsy model. Our results uncover mitochondria as a key regulator of activity set points, demonstrate the differential regulation of set points and compensatory mechanisms, and propose a new strategy to treat epilepsy.

## Highlights

• Inhibition of mitochondrial DHODH stably decreases mean firing rate set point
• DHODH regulates mitochondrial Ca^2+^ buffering and spare respiratory capacity
• Homeostatic responses to activity perturbations are maintained under DHODH blockade
• DHODH inhibition reduces susceptibility to seizures in intractable epilepsy model

## Graphical Abstract

**Figure:**
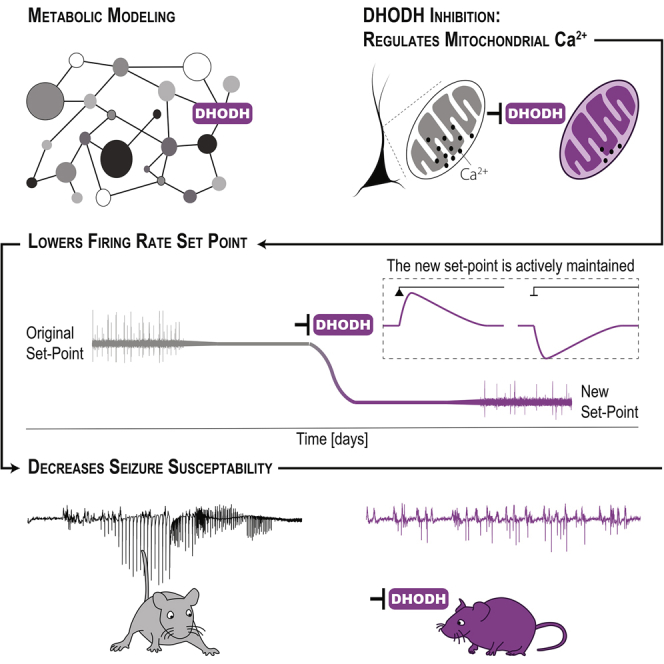

## Teaser

Firing rate set-point regulation has puzzled researchers for decades. Our findings show that mitochondrial DHODH meets the criteria of a bone fide regulator of activity set points and suggest lowering firing set point as a new strategy to treat epilepsy.

## Introduction

How neurons and neural networks maintain average activity levels in a stable regime remains one of the most challenging questions in neuroscience. Since the time of Claude Bernard (Bernard, 1870) and Walter Cannon (Cannon, 1929), scientists have been searching for the core molecular machinery that is at the root of cellular, network, and system-level homeostasis. Although understanding of homeostatic regulation of neural circuits has evolved since its original formulation (Davis, 2006, Marder and Goaillard, 2006, Turrigiano and Nelson, 2004), the core concept encompasses the idea that the internal milieu operates optimally when a regulated physiological variable is kept stable by multiple coordinated mechanisms despite ongoing external or internal variations. A number of models have adopted concepts from engineering control theory to physiological regulation in general (Wiener, 1948) and to neuronal activity regulation in particular (Davis, 2006). According to the control theory, several principle features characterize a system under homeostatic regulation: a set point that defines the output of the system, sensors that detect a deviation from a set point, and effectors that precisely retarget the set point via negative feedback.

Although the concept of a “set point” was proposed by James Hardy 65 years ago (Hardy, 1953–1954) and the existence of an activity set point is widely assumed in neurophysiology, our understanding of set-point establishment is still rudimentary. Accumulated evidence suggests that the mean firing rate (MFR), reflecting an average level of spontaneous spiking activity over extended timescales, represents a physiological variable regulated by homeostatic systems in central neural circuits. Pharmacological, genetic, or experience-dependent perturbations can lead to a rapid change in the MFR that is gradually returned to a set-point level despite the constant presence of a perturbation. This has been shown in cultured neural networks *ex vivo* (Burrone et al., 2002, Slomowitz et al., 2015, Turrigiano et al., 1998, Vertkin et al., 2015) and in primary visual cortex *in vivo* (Hengen et al., 2013, Hengen et al., 2016, Keck et al., 2013). In a given circuit, the same firing properties can arise from a large number of fine-tuned parameters, regulating synaptic and intrinsic membrane properties (Marder and Goaillard, 2006, Prinz et al., 2004). A wide repertoire of homeostatic effector mechanisms that operate at the level of excitatory synapses, inhibitory synapses, and intrinsic excitability enable firing rate renormalization to a circuit-specific MFR set point following perturbations (Davis, 2013, Keck et al., 2017, Maffei and Fontanini, 2009, Pozo and Goda, 2010, Turrigiano, 2011). However, some central questions have remained open. What are the mechanisms that establish the specific values of MFR set points? Are MFR set points fixed (predetermined) or adjustable in central neural circuits? If they are adjustable, do separate mechanisms control negative feedback responses and MFR set-point value? And finally, can re-adjustment of dysregulated firing set points provide a new conceptual way to treat brain disorders associated with aberrant network activity?

We have recently hypothesized that metabolic signaling constitutes a core regulatory module of MFR homeostasis (Frere and Slutsky, 2018). However, the link between neuronal metabolism and MFR homeostasis has remained unexplored. Our *in silico* transcriptome metabolic modeling analysis uncovered mitochondrial dihydroorotate dehydrogenase (DHODH) enzyme as the leading target that rescues metabolic homeostasis of hyperexcitable hippocampal circuits. Using state-of-the-art optical, electrophysiological, and metabolic tools, we identified mitochondria as a central regulator of firing rate set points in hippocampal circuits and DHODH inhibition as a novel strategy to treat epilepsy.

## Results

### Predicting Metabolic Targets that Counteract Chronic Hyperexcitability

**Figure 1:**
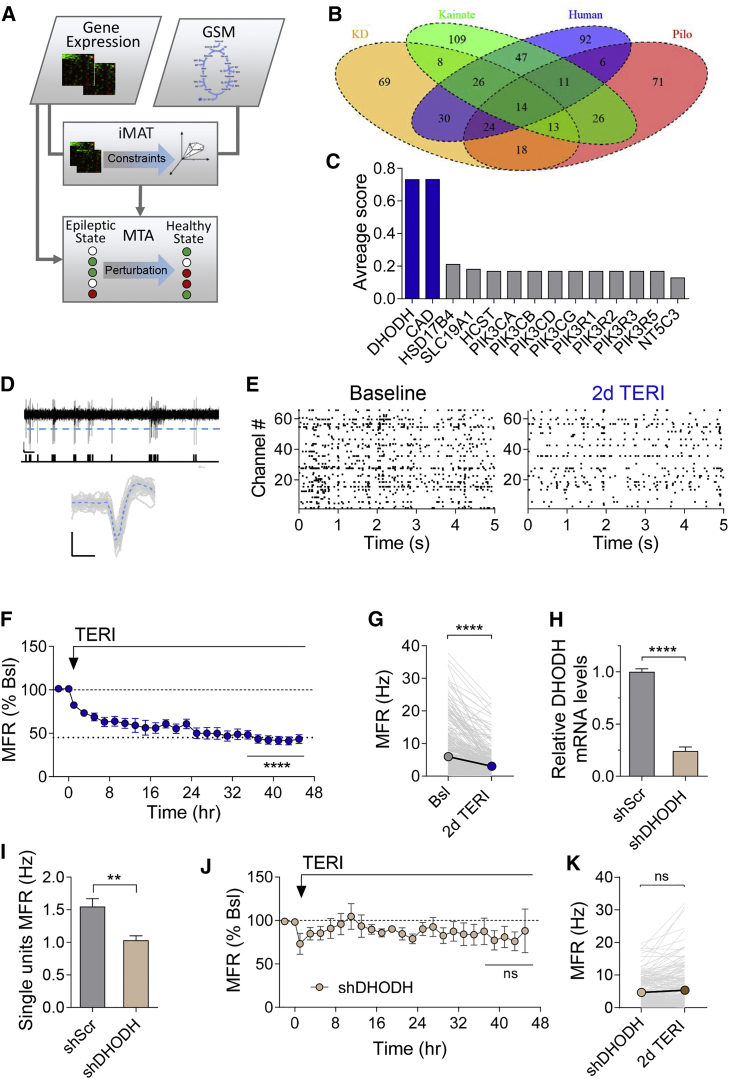
The Leading Computational Prediction, DHODH, Regulates Spontaneous Spiking Rate in Hippocampal Networks (A) Schematic of computational analysis workflow. (B) Diagram showing overlap in genes that pass selection criteria (see STAR Methods) in each test group. Fourteen genes overlapped in all the groups: ketogenic diet (KD; yellow), kainate model (Kainate; green), human idiopathic epilepsy (Human; purple), and pilocarpine model (Pilo; red). (C) Average MTA scores of 14 antiepileptic candidate genes shown in (B). The top candidates, DHODH and upstream CAD enzyme, are shown in blue. (D) Threshold detection of spiking activity from one channel in hippocampal neurons cultured on 120-channel MEA chips. Dotted line denotes threshold; below are the spike time signatures. Scale bars, 20 μV and 50 ms. Bottom: waveforms of spikes extracted from the channel (dotted line denotes average waveform). Scale bars, 20 μV and 1 ms. (E) Raster plots from MEA recordings showing activity from the same 65 channels in baseline and after 2 days of TERI (50 μM) application. (F) The MFR of the network is stably reduced after 50 μM TERI application to 43.28% ± 5.13% of baseline (n = 7 experiments, 504 channels). (G) TERI reduces the average MFR from 5.43 ± 0.32 to 2.46 ± 0.17 Hz (same data as in F). (H) shDHODH reduces DHODH RNA by 75% compared with cells infected with shScr. (I) shDHODH-infected networks (372 single units) display reduced MFR in comparison with shScr-infected ones (299 single units). (J) shDHODH occludes the effect of 50 μM TERI on MFR (n = 5 experiments, 264 channels). (K) TERI (50 μM, 2 days) did not significantly affect MFR per channel following DHODH KD by shDHODH (same data as in J). One-way ANOVA with Dunnett’s multiple comparison test (F and J). Wilcoxon matched-pairs signed rank test (G and K), unpaired Student’s t test (H), and Mann-Whitney test (I). ^∗∗^p < 0.01 and ^∗∗∗∗^p < 0.0001; ns, non-significant (p > 0.05). Error bars represent SEM.

To identify the core molecular targets that regulate metabolic network homeostasis in hippocampal circuits, we used genome-scale metabolic modeling (GSMM; Figure 1A). GSMM has already shown its value in the modeling of human metabolism in health and disease (Duarte et al., 2007, Shlomi et al., 2008, Thiele et al., 2013), including brain metabolism (Lewis et al., 2010). As epilepsy represents a disorder associated with destabilized neuronal activity patterns and metabolic impairments (Lutas and Yellen, 2013, Scharfman, 2015, Zsurka and Kunz, 2015), we hypothesized that a metabolic modeling analysis of epilepsy-associated transcriptome may be useful to predict gene targets linking metabolic and firing homeostasis networks. Accordingly, we analyzed available cortical and hippocampal transcriptome datasets of human epilepsy patients (Delahaye-Duriez et al., 2016), chronic stages of pilocarpine (Okamoto et al., 2010), and kainate (Winden et al., 2011) rat epilepsy models (Table S1). We first integrated the above transcriptome data within the human metabolic model using iMAT (the Integrative Metabolic Analysis Tool) to predict the likely metabolic flux activity in each of the diseases or states mentioned above (Shlomi et al., 2008). The iMAT outputs were subsequently analyzed using a generic metabolic transformation algorithm (MTA), searching for gene perturbations that are most likely to transform a given metabolic state to a desired target one by conducting *in silico* knockout screen of all metabolic genes (Yizhak et al., 2013). That is, in our case we applied the MTA to search for gene perturbations that are most likely to transform the epileptic disease metabolic state back to a healthy one (Figure 1B; Table S3). We found a significant overlap between the MTA predictions and the known seizure-predisposing gene knockouts (Table S2). In addition, our analysis showed a high degree of overlap between prediction set pairs as well as across all analyzed datasets (Figure 1B; Table S4). Specifically, our analysis pointed to the mitochondrial enzyme DHODH as one of the top predicted targets (Figure 1C; Table S3) that transforms toward epilepsy-resistant metabolic state, further confirmed by applying the MTA to the analysis of a ketogenic diet (Table S4; Bough et al., 2006). Hence, we decided to experimentally study the role of DHODH.

### DHODH Inhibition Induces a Stable Reduction in Spontaneous Firing Rates

DHODH is located in the inner membrane of the mitochondria (Jones, 1980) and links two major intracellular processes: the fourth reaction of *de novo* pyrimidine biosynthesis (oxidation of dihydroorotate [DHO] to orotate) and electron transfer from DHO to ubiquinone as part of the mitochondrial electron transport chain (Evans and Guy, 2004; Figure S1A). To explore the role of DHODH in regulation of neuronal activity, we tested the effect of teriflunomide (TERI), a specific, uncompetitive DHODH inhibitor (Bruneau et al., 1998), on the firing properties of high-density hippocampal cultures grown on a multi-electrode array (MEA) for ∼3 weeks. Each MEA contains 120 recording electrodes, each capable of recording the activity of several adjacent neurons (Figure 1D). Spontaneous spiking activity was continuously monitored in an incubator chamber during a baseline recording period and for 2 days following application of 50 μM TERI. TERI application caused a pronounced drop in firing rates (Figures 1E–1G) in a dose-dependent manner (Figure S1B). Surprisingly, the lower MFR induced by TERI was stable during 2 days in the presence of the drug, stabilizing at ∼60% reduction (Figures 1F and 1G). No sign of homeostatic compensation was observed during 2 days of recordings, as expected for typical activity-dependent (Slomowitz et al., 2015, Vertkin et al., 2015) or experience-dependent (Hengen et al., 2013, Hengen et al., 2016, Keck et al., 2013) perturbations. Nevertheless, the effect of TERI on MFR was reversible, as removal of the drug after 2 days restored the original MFR (Figure S1C). Furthermore, TERI efficiently and reversibly inhibited CA3-CA1 synaptic transmission in acute hippocampal slices (half maximal inhibitory concentration [IC_50_] of ∼54 μM) by decreasing the amplitude and the frequency of quantal excitatory synaptic transmission (Figure S2). Thus, reduction in basal excitatory synaptic transmission may contribute to TERI-induced inhibition of spontaneous firing rates.

To verify that TERI’s inhibitory effect on spontaneous spiking activity is mediated via DHODH, we used a lentiviral-based short hairpin (shRNA) delivery methodology to knock down endogenous DHODH. Knockdown (KD) of DHODH by the effective shDHODH (∼80% KD; Figure 1H; Figure S3) caused a reduction of ∼34% in the MFR in comparison with the control hairpin (shScr; Figure 1I) and occluded the inhibitory effect of TERI on MFR (Figures 1J and 1K). On the basis of these results, we conclude that the stable reduction of MFRs by TERI is mediated via DHODH inhibition and not by off targets of the drug.

### TERI Suppresses Spare Mitochondrial Capacity, but Not ATP Production and *De Novo* Pyrimidine Synthesis

**Figure 2:**
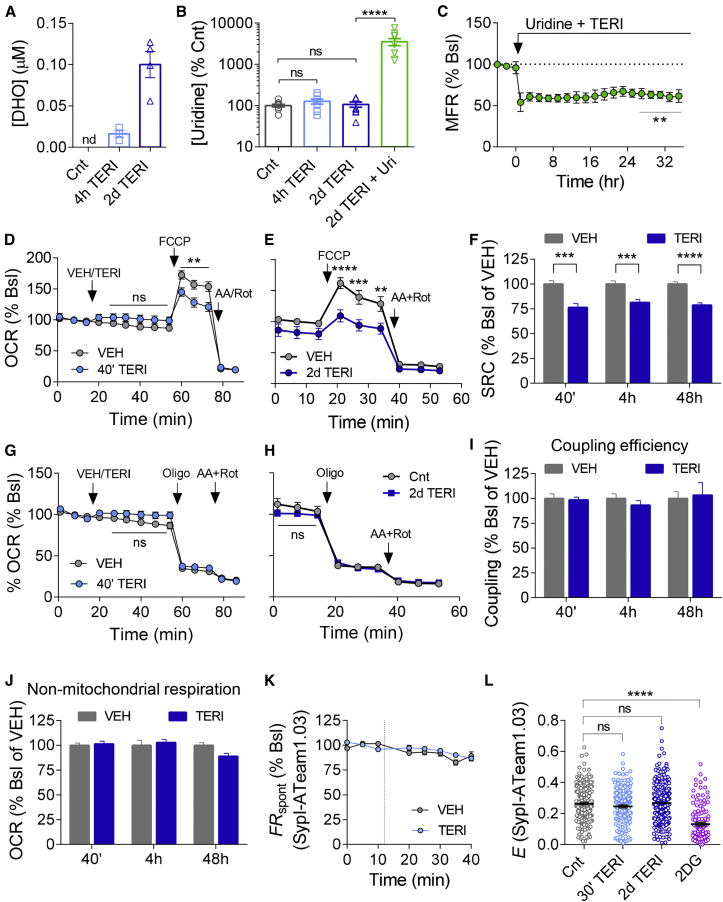
TERI Decreases Spare Respiratory Capacity without Hampering Pyrimidine and ATP Levels (A and B) LC-MS/MS analysis of pyrimidine metabolism. (A) Treatment of hippocampal cultures with 50 μM TERI resulted in a gradual accumulation of intracellular DHO (n = 3 or 4 separate coverslips). DHO levels were non-detectable (nd) in control groups. (B) Intracellular uridine (Uri) levels were not altered by 50 μM TERI application. Addition of exogenous uridine (100 μM) caused an increase in intracellular levels of uridine, indicating that uridine enters the cells (n = 3 experiments, three or four separate coverslips per experiment). (C) Application of 100 μM uridine did not occlude the reduction in MFR by TERI (n = 4 experiments). (D–J) The Seahorse Bioscience XF96 analyzer was used to determine oxygen consumption rates (OCRs) in intact day *in vitro* (DIV) 15 primary hippocampal neurons treated acutely (D) or for 48 h (E) with 100 μM TERI. OCR was monitored for 40 min before determining maximal respiration rates using 3 μM FCCP. Non-mitochondrial oxygen consumption was measured after injection of 1 μM rotenone (Rot) and 2 μg/mL antimycin A (AA). For 2 day TERI treatment, normalization was performed to the baseline of the VEH group. (F) Spare respiratory capacity was stably decreased by TERI (normalized average of three independent experiments, 9–11 wells per condition). (G and H) No difference in ATP-coupled respiration was detected after acute (two independent experiments; 9–12 wells per condition) or 2 day (four independent experiments; 19–17 wells per condition) TERI treatment. (I) The coupling efficiency was not altered by TERI in all time points (p > 0.05, two to four experiments; 8–19 wells per condition). (J) Non-mitochondrial oxygen consumption was not different between the groups (p > 0.05, at least two independent experiments; 8–19 wells per condition). (K) Time course of averaged FRET ratio at synapses of primary hippocampal neurons infected with SypI-ATeam1.03 ATP-FRET sensor (Shulman et al., 2015). No change in the levels of ATP were observed (three independent experiments; three coverslips for vehicle [n = 66 synapses], five coverslips for TERI [n = 187 synapses]). (L) SypI-ATeam1.03 FRET efficiency in synapses was unchanged by acute (30 min, n = 182 synapses) or long-term (2 days, n = 276 synapses) TERI application compared with control (n = 298 synapses). Application of 2-deoxyglucose (2-DG) caused a decrease in FRET efficiency (n = 111 synapses). Two-way ANOVA with Sidak’s multiple-comparisons test (D, E, G, H, and K), one-way ANOVA with Dunnett’s multiple-comparisons test (B, C, and L), and unpaired Student’s t test for each time point (F, I, and J). ^∗∗^p < 0.01, ^∗∗∗^p < 0.001, and ^∗∗∗∗^p < 0.0001; ns, non-significant (p > 0.05). Error bars represent SEM.

DHODH catalyzes the conversion of DHO to orotate in the endogenous synthesis of UMP (Figure S1A). Treatment of hippocampal cultures for 2 days with TERI led to a dramatic accumulation of DHO levels measured by mass spectrometry (Figure 2A), in agreement with inhibition of DHODH enzymatic activity. Notably, 2 days of TERI treatment did not alter the steady-state intracellular levels of uridine (Figure 2B), the end product of *de novo* pyrimidine synthesis, indicating that DHODH is not necessary to maintain normal intracellular levels of uridine in neurons. Moreover, uridine supplementation (100 μM) significantly increased intracellular uridine levels (Figure 2B), without abrogating the inhibitory effect of TERI on MFRs (Figure 2C). Together, these findings demonstrate that stable reduction in firing rates by DHODH inhibition does not depend on pyrimidine levels.

Given that DHODH regulates neuronal activity independently of pyrimidine pathway, we turned to examine the role of the mitochondria in DHODH-mediated neuronal inhibition. Blocking mitochondrial functions by the mitochondrial uncoupler Bam15 (Kenwood et al., 2013), in combination with the ATP synthase blocker oligomycin (to prevent reversal of ATP-ase), inhibited CA3-CA1 synaptic transmission and occluded the effect of TERI (Bam15+Oligo; Figures S4A–S4C). Moreover, Bam15 reduced MFR and occluded the effect of TERI on MFR in MEA recordings (Figure S4D). Addition of Bam15+Oligo after TERI produced an additional inhibition of synaptic transmission (Figures S4E and S4F), indicating that TERI only partially inhibits specific mitochondrial functions, while Bam15 (10 μM) caused mitochondrial uncoupling (Kenwood et al., 2013), nonspecifically impairing several mitochondrial functions.

To directly examine the link between DHODH activity and mitochondria functions, we first set out to assess the effect of TERI on oxygen consumption rate (OCR), an indicator of mitochondrial respiratory activity, using the Seahorse XFe96 analyzer (Qian and Van Houten, 2010). Short-term application of TERI (100 μM) decreased spare respiratory capacity (Figures 2D and 2F), defined as the difference between basal and maximal respiration. Importantly, spare respiratory capacity remained attenuated for 2 days of TERI application (Figures 2E and 2F; Figure S5B). Similar results were obtained with 50 μM TERI (Figures S5A and S5B). Moreover, mtDNA copy number, reflecting mitochondrial mass, remained unaffected following chronic DHODH inhibition (Figure S5C), indicating that the decreased spare respiratory capacity was not the result of a smaller number of mitochondria. In contrast, the coupling efficiency of energy derived from oxidation to oligomycin-sensitive ATP-linked respiration was unaltered following acute and chronic application of TERI (Figures 2G–2I; Figures S5A and S5B). Furthermore, TERI did not affect presynaptic ATP levels, assessed by fluorescence resonance energy transfer (FRET) measurements (Figures 2K and 2L). Non-mitochondrial respiration was not affected at any time point of TERI incubation (Figure 2J).

Taken together, these results indicate that DHODH inhibition stably decreases mitochondrial spare respiratory capacity without inducing compensatory mechanisms. Notably, reduction in spare respiratory capacity did not limit the ability of neurons to fire under high demand, as indicated by an 8.3-fold increase in MFR following blockade of GABA_A_ receptors in TERI-treated neurons (Figure S5D).

### TERI Suppresses Resting Mitochondrial Ca^2+^ Levels while Enhancing Ca^2+^ Transients during Spiking Activity

**Figure 3:**
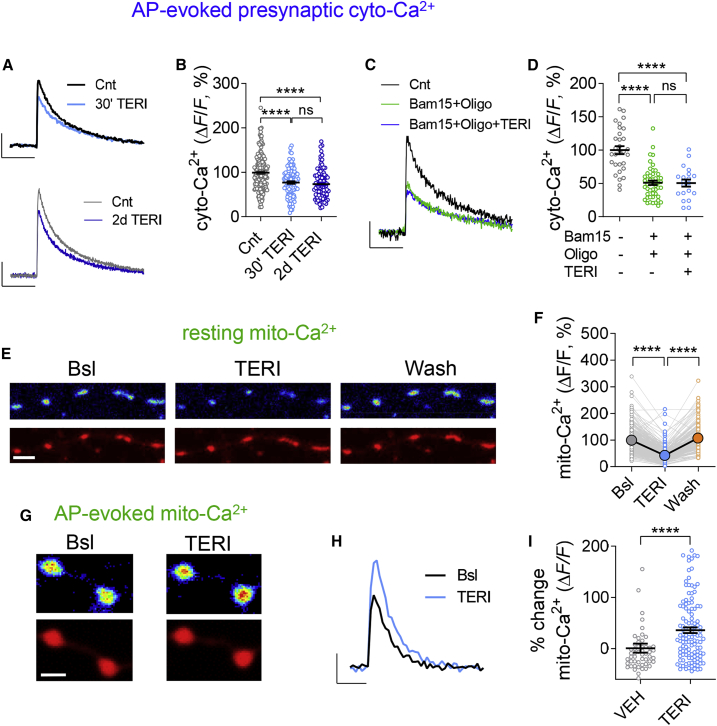
TERI Enhances Mito-Ca^2+^ Buffering during Spiking Activity (A and B) Effect of TERI (50 μM) on AP-evoked presynaptic cyto-Ca^2+^ transients measured by OGB-1AM. (A) Representative traces of Ca^2+^ transients in one experiment evoked by 0.1 Hz stimulation during 500 Hz line scan at boutons and quantified as Δ*F*/*F* (average of ten traces) showing acute (30 min) and long-term (2 days) effect of TERI. Scale bars, 20% and 250 ms. (B) Acute (30 min) and chronic (2 days) TERI reduces the charge transfer of AP-evoked cyto-Ca^2+^ transients (n = 187, 120, and 95 for control, 30 min, and 2 days of TERI, respectively). (C) Representative traces of Ca^2+^ transients in one experiment evoked by 0.1 Hz stimulation during 500 Hz line scan at boutons and quantified as Δ*F*/*F* (average of ten traces) showing acute effect of TERI in the presence of mitochondrial blockers (10 μM Bam15 + 1 μg/mL Oligo). Scale bars, 10% and 250 ms. (D) Application of BAM15+Oligo reduced the charge transfer of AP-evoked cyto-Ca^2+^ transients and occluded the effect of TERI (n = 30, 50, and 20 synapses for control, Bam15+Oligo, and TERI+Bam15+Oligo, respectively). (E) Representative image showing mitochondria co-expressing 2mtGCaMP6m (top) and mCherry-mito (bottom) before, 30 min after TERI addition, and following TERI washout (15 min). Scale bar, 5 μm. (F) Acute (30 min) TERI application reduced resting mito-Ca^2+^ (in the presence of 1 μM TTX to prevent spiking activity) that was restored by washout (n = 330, 330, and 180 mitochondria for control, TERI, and washout, respectively). Large colored dots represent mean. (G–I) Increase in AP-evoked mito-Ca^2+^ transients in the same mitochondria by TERI (50 μM, 30 min). (G) Top: representative 2mtGCaMP6m images showing peak intensity of Ca^2+^ transients evoked by a five AP at 50 Hz burst in the presence of DNQX (2.5 μM) to block recurrent activity, before and after incubation with TERI. Bottom: mCherry-mito expression in the same mitochondria. Scale bar, 2.5 μm. (H) Representative traces of mito-Ca^2+^ transients before (gray) and 30 min after (blue) in one experiment evoked by burst stimulation (5 AP at 50 Hz, inter-burst interval 60 s) during 3 Hz scan at boutons and quantified as Δ*F*/*F* (average of five traces). Scale bars, 100% Δ*F*/*F* and 1 s. (I) Percentage of change in the peak Ca^2+^ transient response by TERI (41.9% ± 7.2% increase, n = 122 mitochondria, six experiments) versus VEH (0.82% ± 8.7%, n = 57 mitochondria, two experiments). One-way ANOVA with Tukey’s multiple-comparisons test (B, D, and F) and Mann-Whitney test (I). ^∗∗∗∗^p < 0.0001; ns, non-significant (p > 0.05). Error bars represent SEM.

Spare respiratory capacity depends on cytosolic (Llorente-Folch et al., 2013) and mitochondrial (Luongo et al., 2015) Ca^2+^ levels. As mitochondria have been recently shown to shape cytosolic Ca^2+^ (cyto-Ca^2+^) in hippocampal terminals (Gazit et al., 2016, Kwon et al., 2016), we tested how DHODH activity affects presynaptic Ca^2+^ buffering. Acute TERI application caused a decrease in action potential (AP)-induced presynaptic Ca^2+^ transients measured by Oregon green 488 BAPTA-1 AM (OGB-1 AM) that remained stable for 2 days (Figures 3A and 3B). This was accompanied by a reversible increase in resting cyto-Ca^2+^ levels (Figures S6A and S6B). Mitochondrial blockers (Bam15+Oligo) decreased presynaptic Ca^2+^ transients and occluded the effect of TERI (Figures 3C and 3D), implicating mitochondrial DHODH in regulation of the presynaptic Ca^2+^ transients. To directly assess the role of mitochondria in regulation of Ca^2+^ transients, we measured the effect of TERI on mitochondrial Ca^2+^ (mito-Ca^2+^) using the highly sensitive, genetically encoded Ca^2+^ probe GCaMP6m (Chen et al., 2013) efficiently targeted to mitochondria (2mtGCaMP6m; Gazit et al., 2016). 2mtGCaMP6m colocalized with the mitochondrial marker (mCherry-mito) in hippocampal neurons (Figure S6C). Indeed, application of TERI caused a reversible ∼60% decrease in resting mito-Ca^2+^ (Figures 3E and 3F; Figure S6D). This TERI-induced reduction in resting mito-Ca^2+^ was accompanied by an increase in AP-evoked mito-Ca^2+^ for bursts (five APs at 50 Hz; Figures 3G–3I) and for single APs (Figure S6E). Taken together, these results suggest that DHODH inhibition suppresses AP-evoked cyto-Ca^2+^ transients by facilitating mito-Ca^2+^ buffering during spiking activity at hippocampal boutons.

### DHODH Inhibition Stably Decreases Intrinsic Excitability, mEPSC Amplitude, and Frequency

**Figure 4:**
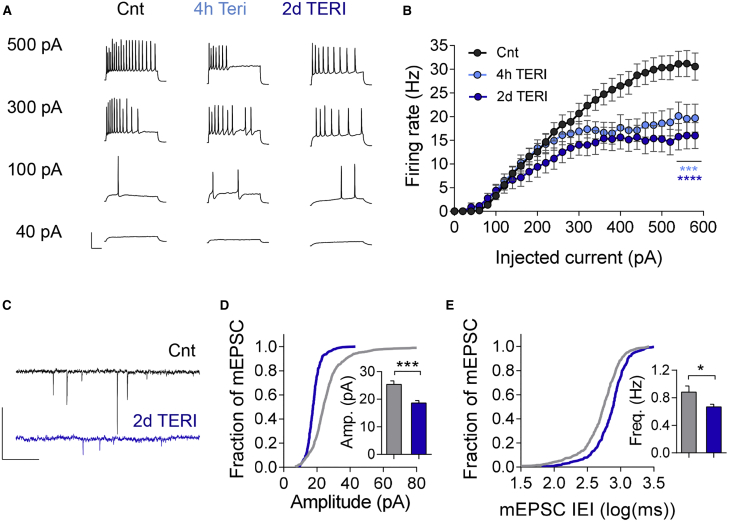
DHODH Inhibition Reduces Intrinsic Excitability and mEPSC (A) Representative traces of voltage responses evoked by 20 pA step of current injections in control, after 4 h and 2 day TERI incubation in hippocampal cultures. Scale bars, 40 mV and 100 ms. (B) F-I relationship. After 4 h and 2 days of incubation, there was a significant attenuation of spiking rates at higher current injections, resulting in lower maximal firing rates. n = 27, 20, and 16 for control, 4 h, and 2 days, respectively. (C) Representative traces of mEPSCs for control and 2 day TERI incubation. Scale bars, 40 pA and 1 s. (D) Cumulative histograms of mEPSC amplitudes in control (n = 14) and following 2 day TERI incubation (n = 17). Inset: TERI reduced mean mEPSC amplitude from 25.4 pA in control to 18.7 pA following 2 days of application. (E) Cumulative histogram of mEPSC inter-event intervals showing larger intervals after 2 day TERI incubation (same experiments as in D). Inset: TERI reduced mean mEPSC frequency after 2 days of incubation (p = 0.024). Two-way ANOVA with Tukey’s multiple-comparisons test (B) and Student’s t test (D and E). ^∗^p < 0.05, ^∗∗∗^p < 0.001, and ^∗∗∗∗^p < 0.0001. Error bars represent SEM in all graphs.

What are the functional rearrangements in network activity that underlie sustained reduction in spontaneous firing? To answer this question, we first assessed whether intrinsic excitability is changed after DHODH inhibition. We elicited APs in response to increasing somatic current injections ranging from 0 to +600 pA (F-I curves) in the presence of postsynaptic receptor blockers (Figure 4A). TERI elicited a reduction in response to higher current injections, resulting in a sharp reduction in the maximal firing frequency after 4 h that remained low during 2 days (Figures 4A and 4B). Moreover, DHODH KD occluded the effect of TERI (Figures S7A and S7B), confirming that reduction in maximal firing frequency by TERI is mediated by DHODH. Input resistance, AP threshold, and AP width and amplitude showed no significant change following TERI application (Figures S7C–S7F). Next, we examined the effect of DHODH inhibition on adaptions of excitatory synapses. TERI induced a pronounced reduction in miniature excitatory postsynaptic current (mEPSC) amplitude following 2 days of incubation (Figures 4C and 4D) and significant, but moderate, reduction in mEPSC frequency (Figures 4C and 4E). These data indicate that DHODH inhibition does not activate typical compensatory mechanisms, leading to the sustained reduction in MFRs.

### TERI Regulates MFR Set Point in Hippocampal Networks

**Figure 5:**
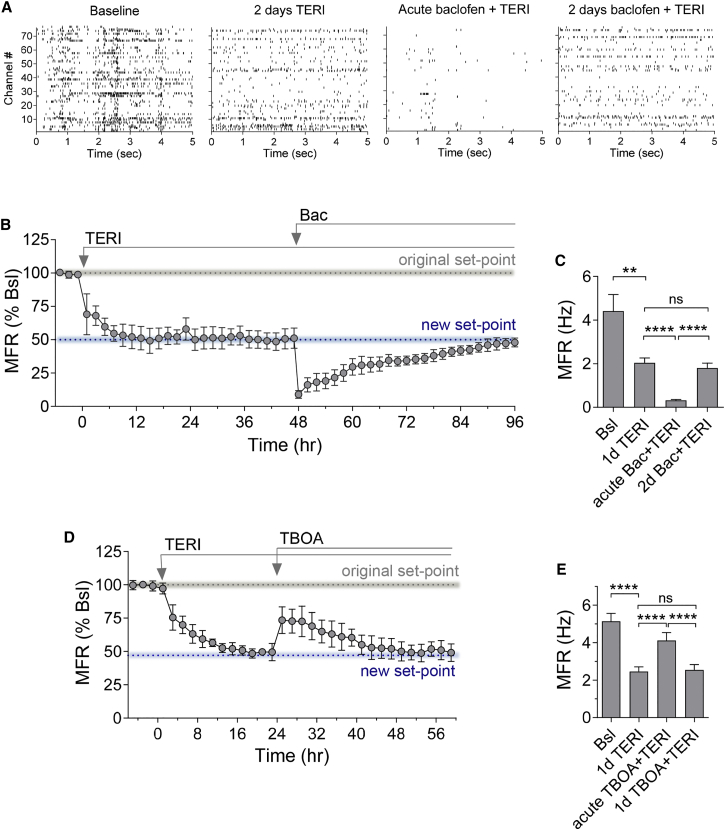
DHODH Regulates MFR Set Points Independently of Compensatory Feedback Mechanisms (A) Raster plots showing reduction of MFR after 2 days of 50 μM TERI application, acute reduction of MFR after 10 μM baclofen application in the presence of TERI, and recovery of spiking activity after 2 more days in the presence of both TERI and baclofen. (B) Time course of MFR reduction after TERI and renormalization of MFR after baclofen to a new, lower set point (n = 4 experiments, 270 channels). (C) Average MFR data across 270 channels (same experiments as in B). (D) Time course of MFR reduction after TERI and renormalization of MFR after TBOA back to the new, lower set point (n = 4 experiments, 299 channels). (E) Average MFR data across 299 channels (same experiments as in D). One-way ANOVA with Tukey’s multiple-comparisons test (C and E). ^∗∗^p < 0.01 and ^∗∗∗∗^p < 0.0001; ns, non-significant (p > 0.05). Error bars represent SEM in all graphs.

These results raise the question of whether feedback regulation mechanisms are completely disabled by DHODH inhibition. If so, the network will be incapable of regulating its own activity against future perturbations. Another possibility is that regulatory feedback mechanisms are still active, yet they are “tuned” to a lower level of activity. In this scenario, further perturbations will induce compensatory responses to sustain its current low activity state (Styr and Slutsky, 2018). However, whether firing set points are adjustable and whether compensatory responses and the set points are separately controlled is unknown. Our previous work has demonstrated a renormalization of the MFR to a set-point level following firing inhibition by a GABA_B_ receptor agonist baclofen through intrinsic and synaptic adaptive mechanisms (Slomowitz et al., 2015). Here, we used this assay to determine whether activity-dependent compensatory feedback mechanisms are still active under DHODH inhibition. Therefore, we added 10 μM baclofen following 2 days of incubation with TERI (Figures 5A–5C). Addition of baclofen in the presence of TERI induced a transient reduction of the MFR that was gradually corrected over a 2 day period. The adaption stabilized at the new, lower steady-state level established following TERI application (Figures 5A and 5B). The lower set point was reestablished in each network according to its own steady state (Figures S8A and S8B), as well as on average across all the experiments (Figure 5C). Importantly, partial mitochondrial uncoupling by low concentration of Bam15 (1 μM) caused a stable, ∼42% reduction in the MFR but impaired MFR renormalization following baclofen application (Figures S8C–S8E). These experiments indicate that inhibition of specific, DHODH-dependent mitochondrial functions is critical for lowering MFR set point without impairing homeostatic feedback responses.

If DHODH is a bona fide regulator of activity set points, MFR renormalization to a new set-point value should occur for bi-directional changes in activity. To test this prediction, we induced chronic hyperactivity in DHODH-inhibited neurons by enhancing glutamate spillover via inhibition of glutamate transporters (Asztely et al., 1997). Notably, addition of 10 μM TBOA, a subsaturating concentration of the competitive glutamate transporter antagonist (Christie and Jahr, 2006), following 1 day of incubation with TERI induced a transient increase in MFR that was gradually renormalized during the following day to the new set-point value (Figures 5D and 5E). These results demonstrate that homeostatic compensation mechanisms are still active under DHODH inhibition yet are tuned to drive the steady state of the network to a new, lower set-point level for bi-directional activity perturbations.

### TERI Reduces MFR and Synaptic Transmission in the Hippocampus *In Vivo*

**Figure 6:**
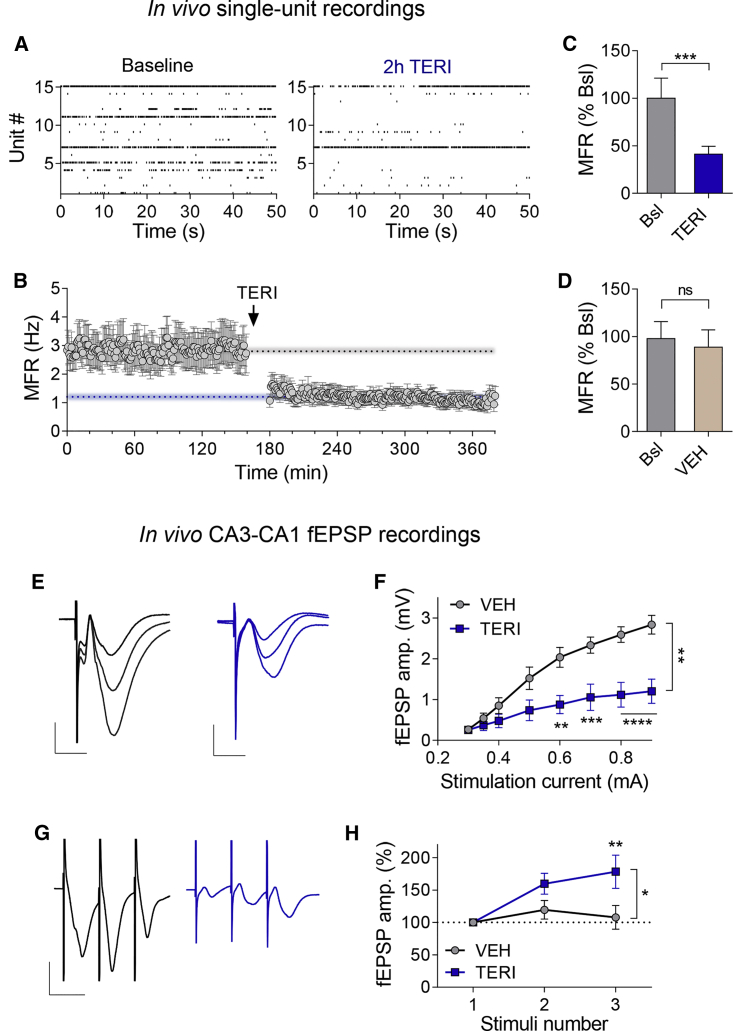
TERI Suppresses MFR in the CA1 and the CA3-CA1 Synaptic Transmission *In Vivo* (A–D) Single-unit recordings in the CA1 following a single i.c.v. injection of TERI (1 μL, 27 μg) or vehicle (VEH; 1 μL). (A) Representative raster plots demonstrating single-unit spiking activity during baseline (left) and following i.c.v injection of TERI (right). (B) Single-unit MFR during baseline recording and following TERI administration (seven mice, 92 single units); dotted gray line represents the mean value during baseline (2.9 Hz), and dotted blue line represents mean value after TERI administration (1.2 Hz). (C) MFR in baseline and after TERI administration (same data as in B). (D) MFR in baseline and after VEH administration (same data as in Figure S9D). (E–H) TERI (1 μL, 27 μg) or the same volume of vehicle (VEH) was injected i.c.v. daily for 3 consecutive days. fEPSP recordings were done 2–4 h after the last injection. (E) Representative traces of fEPSP after TERI (blue) or VEH (gray) administration evoked by 0.5, 0.6, and 0.7 mA stimulation. Scale bars, 0.5 mV and 10 ms. (F) Response of fEPSP amplitude to increased current injection shows an attenuation after treatment with TERI (n = 6 mice) compared with VEH (n = 6 mice). (G) Representative traces of fEPSP evoked by 3 stimuli at 50 Hz in TERI (blue) versus VEH (gray) treated mice. Scale bars, 1 mv and 20 ms. (H) fEPSP amplitude normalized to the first response during a burst stimulation (three stimuli, 50 Hz) shows increased facilitation after treatment with TERI (n = 9 mice) compared with VEH (n = 9 mice). Mann-Whitney nonparametric test (C and D) and two-way ANOVA with Sidak’s multiple-comparisons test (F and H). ^∗^p < 0.05, ^∗∗^p < 0.01, ^∗∗∗^p < 0.001, and ^∗∗∗∗^p < 0.0001; ns, non-significant (p > 0.05). Error bars represent SEM.

Given a profound difference in energy metabolism between *in vivo* and *ex vivo* central neuronal networks, we asked whether DHODH inhibition modulates spiking activity *in vivo*. Because TERI does not cross the blood-brain barrier efficiently (Vidal-Jordana et al., 2015), we performed intracerebroventricular (i.c.v.) infusion of TERI (27 μg in 1 μL) versus vehicle (VEH; 1 μL). We recorded single-unit activity in behaving adult mice with chronically implanted tetrodes (Figures S9A–S9C). Compared with baseline, i.c.v. TERI injection caused a stable decrease of ∼60% in the MFR of CA1 neurons (Figures 6A–6C). In contrast, similar amount of VEH did not affect the MFR across several hours of recording (Figure 6D; Figure S9D). These results reveal an inhibitory effect of TERI on spontaneous spiking activity in the hippocampus of behaving mice.

Next, we injected TERI versus VEH daily for 3 consecutive days, and recorded field excitatory postsynaptic potentials (fEPSPs) in the CA3-CA1 pathway in anesthetized mice 2–4 h after the last TERI injection. Strikingly, the slope of input (stimulation current) to output (fEPSP amplitude) was reduced by ∼64% following i.c.v. injections of TERI (Figures 6E and 6F). Furthermore, TERI increased short-term synaptic facilitation during high-frequency stimulation (Figures 6G and 6H), similarly to its effect in acute hippocampal slices (Figure S2), indicating inhibition of glutamate release probability (Dobrunz and Stevens, 1997) at CA3-CA1 synapses.

### TERI Reduces the Susceptibility to Seizures in Acute PTZ Model

**Figure 7:**
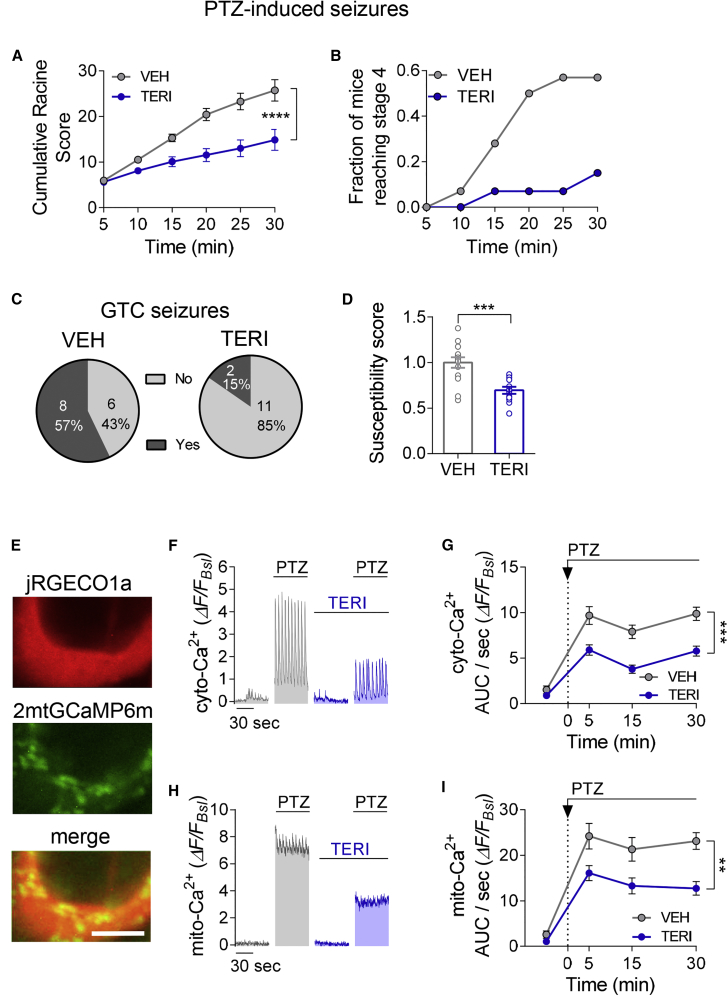
TERI Reduces Susceptibility to PTZ-Induced Seizures (A–D) Effect of TERI on PTZ epilepsy model. TERI (1 μL, 27 μg) or the same volume of vehicle (VEH) was injected i.c.v. daily for 3 consecutive days. PTZ (70 mg/kg) was injected intraperitoneally (i.p.) 2–4 h after the last i.c.v. injection of TERI or VEH. TERI displays potent antiepileptic effects as demonstrated by lower cumulative Racine score (A), reduced number of mice reaching stage 4 (B), smaller number of GTC seizures (C), and reduced susceptibility to seizure induction (D). TERI (blue; n = 13) versus VEH (gray; n = 14) treated mice. (E–I) TERI pre-incubation attenuates PTZ effect on cyto- and mito- Ca^2+^ in hippocampal cultures. Neurons were infected with 2mt-GCaMP6m and jRGECO1 to follow mito- and cyto-Ca^2+^, respectively, in the same cell during spontaneous activity. TERI reduces Ca^2+^ overload in the cytosol (F and G) and in the mitochondria (H and I) following PTZ application in neuronal cultures. (E) Representative images of a neuron co-expressing the cyto-Ca^2+^ sensor jRGECO1a (top, red) and the mito-Ca^2+^ sensor 2mtGCaMP6m (middle, green), and the merged image (bottom). Scale bar,: 5 μm. (F) Representative traces of two neurons showing cyto-Ca^2^ activity before and 15 min after application of PTZ (10 mM). Blue, pre-incubated with TERI; gray, pre-incubated with VEH. (G) Summary of cyto-Ca^2+^ level at 15 min intervals during 30 min of recording following PTZ application. Neurons pre-incubated with TERI (n = 29) had lower cyto-Ca^2+^ levels compared with those with VEH (n = 18). (H) Representative traces showing the effect of PTZ on mito-Ca^2+^ (analysis of the same cells as in E). PTZ caused Ca^2+^ overload in the mitochondria. This effect was attenuated in neurons pre-incubated with TERI. (I) Summary of total mito-Ca^2+^ level at 15 min intervals during 30 min of recording following PTZ application (same cells as in G). Neurons pre-incubated with TERI (n = 29) had lower cyto-Ca^2+^ levels compared with those with VEH (n = 18). Mann-Whitney nonparametric test (D) and two-way ANOVA with post hoc Sidak’s tests (A, G, and I) were used for the analysis. ^∗∗^p < 0.01, ^∗∗∗^p < 0.001, and ^∗∗∗∗^p < 0.0001. Error bars represent SEM.

Given that DHODH is the leading computation prediction of the metabolic modeling analysis based on hippocampal transcriptome of epilepsy patients and rodent epilepsy models, we asked whether i.c.v. injection of TERI regulates the susceptibility of mice to seizures. First, we used a pentylenetetrazole (PTZ) mouse model, one of the most widely used animal seizure models in the search for new antiepileptic drugs (Bialer and White, 2010). Pretreatment with i.c.v. injections of TERI for 3 days significantly reduced the cumulative PTZ revised Racine scores (Figure 7A) and the fraction of mice reaching Racine stage 4 at any point during the first 30 min following PTZ injection (Figure 7B). Furthermore, the fraction of mice that developed generalized tonic-clonic (GTC) seizures was reduced from 57% to 15% by TERI treatment (Figure 7C), as well as seizure susceptibility (Figure 7D). These results demonstrate antiepileptic protection by cerebral DHODH inhibition against induced seizures in an acute PTZ model of epilepsy.

**Figure 8:**
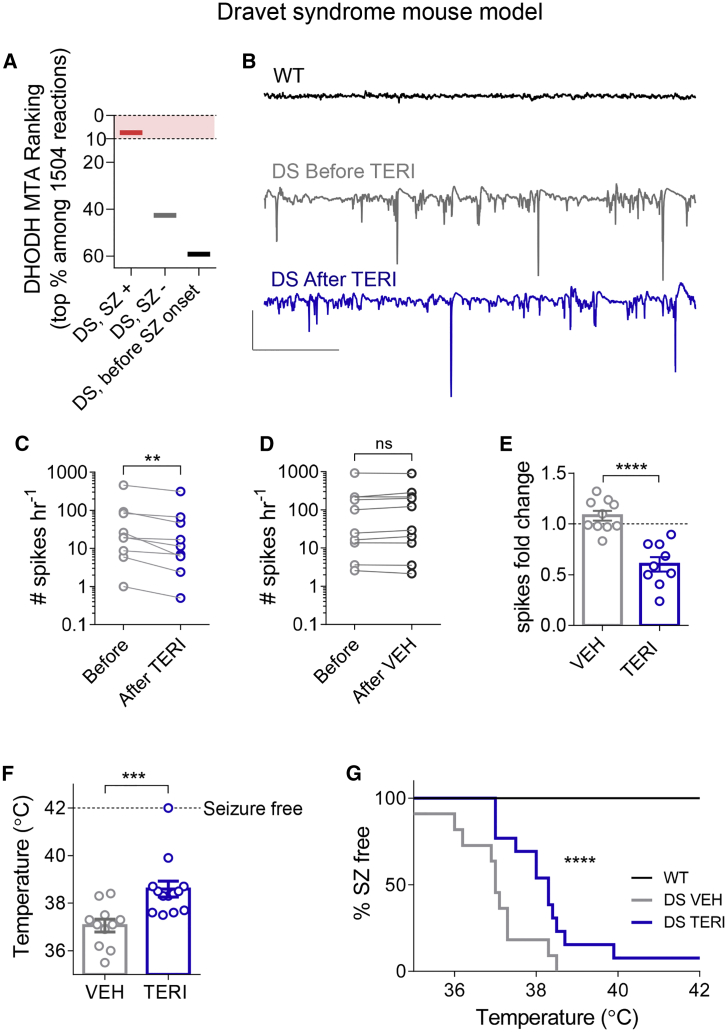
TERI Reduces the Frequency of Interictal Spikes and the Susceptibility to Thermally Induced Seizures in the Mouse Model of DS (A) MTA analysis of hippocampal RNA sequencing data in DS model *Scn1a*^+/−^ mice (Hawkins et al., 2019). DHODH was ranked within top 10% among analyzed 1,504 metabolic reactions in DS mice with seizures (red line, P24 SZ+ group), but only at 43% in DS mouse strain without seizures (gray line, P24 SZ− group) and at 59% in DS mice before seizures onset (black line, P14 group). (B) Representative traces of LFP recordings in the CA1 stratum radiatum depict normal activity in WT (top trace), interictal activity before i.c.v. injection of TERI (middle trace), and after injection (bottom trace) in DS model mice. Scale bars, 5 mv and 20 s. (C) TERI i.c.v. injection caused a significant reduction in the frequency of interictal spikes (n = 9 mice, p = 0.039). (D) VEH i.c.v. injections did not affect the frequency of interictal spikes (n = 10 mice, p = 0.49). (E) On average, the TERI-treated group displayed a 1.8-fold reduction in the frequency of interictal spikes (same data as in B and C). (F) Repeated i.c.v. injections of TERI for 3 consecutive days increased temperature threshold of thermally induced seizures. Temperature threshold for seizure induction was 37.05°C ± 0.26°C in VEH-treated group (n = 11 mice) and 38.59°C ± 0.33°C in the TERI-treated group (n = 13 mice). (G) Percentage of mice remaining free of behavioral seizures (SZ) at the indicated body core temperatures. All WT mice (n = 8) remained SZ free during the experiment. Wilcoxon matched-pairs (C and D), Mann-Whitney nonparametric test (E and F), and log rank (Mantel-Cox) test (G) were used for the analysis. ^∗∗^p < 0.01, ^∗∗∗^p < 0.001, and ^∗∗∗∗^p < 0.0001. Error bars represent SEM.

To test how DHODH inhibition regulates cyto- and mito-Ca^2+^ levels in hyperexcitable hippocampal neurons, we analyzed the effect of PTZ in VEH- versus TERI-pretreated neuronal cultures. We performed time-lapse, simultaneous imaging of cyto- and mito-Ca^2+^ transients in somata of hippocampal neurons before and after PTZ application. Ca^2+^ dynamics in the cytosol were measured using a red-shifted jRGECO1a sensor and Ca^2+^ in mitochondria using 2mtGCaMP6m. Application of PTZ to cultured hippocampal neurons triggered a massive increase in both mito-Ca^2+^ and cyto-Ca^2+^ (Figures 7F–8I; Figure S10A). Accumulation of mito-Ca^2+^ by PTZ was dependent on an increase in spiking activity, as TTX abolished PTZ-induced mito-Ca^2+^ augmentation (Figure S10B). Indeed, high-frequency electrical stimulation, mimicking PTZ-induced spontaneous bursts, also triggered an accumulation of mito-Ca^2+^, accompanied by a decrease in the amplitude of mito-Ca^2+^ responses (Figure S10C). Moreover, increases in cyto-Ca^2+^ and mito-Ca^2+^ levels were highly correlated per neuron following PTZ application (Figure S10D). We also obtained similar results using a lower affinity probe (mtRCaMP1h; Figure S10E), suggesting that these results are not due to probe saturation and have a biological origin. As expected from inhibitory long-term effects of TERI on intrinsic neuronal excitability and on synaptic excitatory currents (Figure 4), TERI preincubation diminished cyto-Ca^2+^ accumulation by PTZ (Figures 7F and 7G). Thus, in TERI-treated neurons, lower cyto-Ca^2+^ events resulted in attenuated mito-Ca^2+^ levels at different time points of PTZ application (Figures 7H and 7I), probably because of smaller Ca^2+^ fluxes into mitochondria. Because mito-Ca^2+^ overload has been associated with seizure activity (Folbergrová and Kunz, 2012) and neuronal death (Orrenius et al., 2003), decreased Ca^2+^ accumulation in the mitochondria may contribute to antiepileptic effects of TERI.

### TERI Reduces the Susceptibility to Seizures in Dravet Syndrome Model

Finally, we decided to assess the efficiency of TERI in a genetic mouse model of Dravet syndrome (DS), one of the most intractable and severe forms of childhood epilepsy, largely resistant to current antiepileptic medications (Wirrell et al., 2017). Epilepsy in DS patients begins in the first year of life with febrile seizures that progress to refractory seizures and frequent episodes of status epilepticus (Dravet, 2011). More than 80% of patients diagnosed with DS carry a *de novo* mutation within the voltage-gated sodium channel gene *SCN1A* (Catterall et al., 2010, Zuberi et al., 2011).

Experimental mouse models of DS, based on *Scn1a* mutations, are an exceptional genocopy and phenocopy of the human disease. Similarly to DS patients, DS mice are asymptomatic during their first weeks life and start experiencing spontaneous seizure toward their fourth week (Ogiwara et al., 2013, Yu et al., 2006).

First, we applied metabolic modeling to the recently published hippocampal RNA sequencing data from the DS model *Scn1a*^+/−^ mice (Hawkins et al., 2019). Our MTA results demonstrate that DHODH was ranked in the top 10% of candidates (7.3%) among DS after the onset of spontaneous seizures, but not in DS mice before the onset of spontaneous seizures and not in DS mice lacking spontaneous seizures (Figure 8A). To test whether DHODH inhibition indeed provides protection to adult DS mice, we used a knockin DS mouse model carrying the *Scn1a*-A1783V mutation (Alonso Gómez et al., 2018). Similarly to other DS mouse models, these mice exhibited thermally induced seizures and profound premature death, with less than 40% that survive beyond the sixth week of life (Figure S11A). DS mice displayed frequent spontaneous interictal spikes in the CA1 stratum radiatum that were not evident in wild-type (WT) mice (Figure 8B). Significant reduction in the frequency of interictal spikes was observed 2–4 h after i.c.v. injection of TERI (Figure 8C), whereas VEH injections did not affect interictal spikes frequency (Figure 8D). As a result, the TERI-treated group displayed a 1.8-fold reduction in the frequency of interictal spikes (Figure 8F).

Next, we tested how repeated i.c.v. injections of TERI versus VEH for 3 consecutive days affect the susceptibility to thermally induced seizures. The temperature threshold for seizure induction was 37.05 ± 0.26°C in the VEH-treated group, compared with 38.59°C ± 0.33°C in TERI-treated group (Figures 8F and 8G). Thus, TERI decreased the susceptibility for thermally induced seizures in DS mice. Altogether, these results demonstrate that cerebral DHODH inhibition conveys potent antiepileptic protection even in the severe, pharmacoresistant genetic model of epilepsy.

## Discussion

The present work supports our recent hypothesis suggesting that mitochondrial signaling is a core member of homeostatic machinery underlying firing homeostasis (Frere and Slutsky, 2018). These results are important for several reasons. First, they uncover previously unrecognized mechanisms regulating excitatory synaptic transmission, neuronal excitability, and spontaneous firing by basal DHODH activity. Second, they provide a proof of principle of the mitochondrial role in the regulation of activity set points. Third, they reveal that activity set points and homeostatic compensatory mechanisms are separately regulated in neuronal networks. Fourth, they reveal a new potential strategy to suppress seizures. Fifth, they demonstrate the power of genome-wide computational approaches to predict homeostatic regulators in specific neural circuits.

### MFR Set-Point Characterization

Set points in physiology have been a puzzle for decades, leaving many central questions unanswered: Do set points exist? Is the set point fixed or variable? What are the mechanisms underlying a set-point establishment? In this study, we developed an experimental framework to identify the core regulators of MFR set points versus compensatory effector mechanisms in neural circuits. Our results show that DHODH met the criteria of bona fide regulator of set-point establishment: (1) the perturbation causes a stable change in the controlled variable without inducing a compensatory response, and (2) known, bi-directional activity perturbations that induce firing renormalization under control conditions still activate compensatory mechanisms but toward the new set-point value (Styr and Slutsky, 2018). On the basis of our data, we conclude that firing set points are tunable in hippocampal networks. Thus, in addition to many physiological variables related to energy metabolism, such as adiposity, insulin resistance, and glucose and lipid homeostasis, firing rate homeostasis is characterized by adjustable set points. In comparison with fixed set points, adjustable set points have been assumed to provide a greater degree of adaptability but are more vulnerable to dysregulation, yielding chronic diseases (Kotas and Medzhitov, 2015).

Another important conclusion based on our results relates to the differential regulation of homeostatic feedback responses and firing rate set points. Although several candidates have been proposed to be required for specific adaptive mechanisms, the master regulator orchestrating multiple homeostatic responses remains to be uncovered. It is worth mentioning that general reduction in the mitochondrial membrane potential impairs homeostatic responses to activity perturbations, suggesting that DHODH regulates activity set points via specific mitochondrial signaling pathways. Whether a lower MFR set point imposed by DHODH inhibition is caused by a modulation of a putative Ca^2+^ sensor or by readjusting the expression of homeostatic effectors remains to be elucidated. Future studies are needed to identify the cellular program underlying activity set-point regulation by mitochondrial DHODH in central mammalian circuits.

### DHODH as a Link between Firing, Ca^2+^, and Metabolic Homeostasis

How does DHODH activity regulate mitochondrial functions in hippocampal neurons? Mitochondria play a critical role in energy metabolism and Ca^2+^ homeostasis in neurons (Devine and Kittler, 2018). Both Ca^2+^ buffering and ATP production have been shown to profoundly affect presynaptic function (Gazit et al., 2016, Kwon et al., 2016, Rangaraju et al., 2014, Vaccaro et al., 2016, Verstreken et al., 2005). Although DHODH inhibition does not alter ATP levels, it diminishes spare respiratory capacity of mitochondria in hippocampal neurons. Given the dependency of spare and maximal respiratory capacity on cyto- and mito-Ca^2+^ (Llorente-Folch et al., 2013, Luongo et al., 2015), these metabolic changes may be caused by DHODH-mediated modifications in Ca^2+^ homeostasis. Namely, TERI profoundly and rapidly decreases resting mito-Ca^2+^, while enhancing Ca^2+^ buffering during spiking activity. Thus, DHODH links metabolic, Ca^2+^, and firing homeostasis.

How do hippocampal neurons respond to DHODH-mediated changes in mitochondrial functions? The most intuitive explanation of the observed stable inhibition of spiking network activity would be that neuronal activity is limited by diminished spare respiratory capacity. However, the hippocampal network can fire at 8-fold higher rates under DHODH inhibition. Thus, instead of limiting MFR, decreased spare respiratory capacity constitutes the regulatory MFR mechanisms. Although previous studies linked decreased spare respiratory capacity to pathological conditions (Nicholls, 2008), lowering the MFR set point may present an adaptive strategy of neurons in response to diminished spare respiratory capacity. Such an adaptive decrease in spiking activity and a subsequent reduction in energy usage may serve to maintain metabolic homeostasis and long-term tissue viability in hippocampal circuits. It is worth noting that short-term synaptic facilitation was even enhanced following chronic DHODH inhibition *in vivo*. Thus, limiting the spare respiratory capacity of mitochondria results in lowering the MFR set point, without sacrificing input-specific information processing and homeostatic plasticity to persistent activity changes.

### Activity Set Points and Epilepsy

Neuronal activity, being heavily dependent on glucose supply as the main fuel source (Magistretti and Allaman, 2015), is especially vulnerable to metabolic dysregulation. Thus, pathological brain states characterized by aberrant firing are likely to be associated with a dysregulated metabolic network. Indeed, mitochondrial dysfunctions have been linked to initiation and progression of distinct types of epilepsy (Zsurka and Kunz, 2015), a brain disorder characterized by firing instability and recurrent seizures, reflecting aberrant synchronous activity of large groups of neurons.

Our results highlight the potential of cerebral DHODH as a therapeutic target for epilepsy. Previous studies extensively explored DHODH as a potential drug target across autoimmune diseases, oncology, and infectious diseases. TERI is approved for multiple sclerosis treatment and presumably acts by antagonizing the proliferation of lymphocytes via *de novo* pyrimidine synthesis (Bar-Or, 2014). However, our results demonstrate that in terminally differentiated neurons, TERI attenuates spontaneous spiking activity via regulation of mitochondrial functions. Our results suggest that TERI is likely to suppress hyperexcitability by mitigating mito-Ca^2+^ overload. This mechanism of action is different from the known metabolic antiepileptic strategies such as a ketogenic diet (Peterman, 1925) and stiripentol (Sada et al., 2015) that are thought to primarily act on the availability of energy fuel. Given that cortical hyperactivity is associated with the remission phase of multiple sclerosis in a mouse model (Ellwardt et al., 2018), reduction of firing set point by cerebral DHODH inhibition may provide a new way to slow down neurodegeneration.

At least 25%–40% of epilepsy patients are resistant to drug therapy and do not receive adequate seizure control (Schmidt and Sillanpää, 2012). DS is among the most drug-resistant forms of epilepsy (Dravet, 2011). Drugs currently used as first-line therapy for Dravet patients, such as valproate and clobazam, have limited efficacy, and other antiepileptic drugs, including lamotrigine, vigabatrin, and oxcarbazepine, even exacerbate seizures in Dravet patients (Ziobro et al., 2018). Alternative new therapies are urgently needed for this highly refractory epilepsy syndrome. In this study, we demonstrated beneficial effects of TERI in a mouse model of DS, including markedly reduced occurrence of interictal spikes and an increase of 1.5°C in the susceptibility to thermally induced seizures (Figure 8E). Of note, a difference of 1.2°C was observed between DS mice on the C57BL/6 genetic background, which exhibit more than 60% premature mortality, and DS mice on the pure 129/SvJ genetic background, which have mild epilepsy and about 90% survival (Rubinstein et al., 2015, Yu et al., 2006).

In light of our findings, the therapeutic potential of cerebral DHODH inhibition deserves to be further explored with regard to intractable epilepsy. On the basis of our data, we conclude that mild mitochondrial inhibition can be used for therapeutic purposes only if homeostatic feedback responses to ongoing perturbations and information processing remain uncompromised. Our work provides the foundation for future studies on the mechanisms by which mitochondria adjust activity set points and on the role of dysregulated set points in the pathophysiology of epilepsy and perhaps other disorders associated with aberrant network activity.

## STAR★Methods

### Key Resources Table

**Table:** 

REAGENT or RESOURCE	SOURCE	IDENTIFIER
**Bacterial and Virus Strains**		

pAAV-hSyn-2mtGCaMP6m	This paper	N/A
pAAV-hSyn-mCherry-mito	This paper	N/A
pAAV-hSyn- jRGECO1a	This paper	N/A
pAAV-CBAP-SypI-ATeam1.03	Shulman et al., 2015	N/A
pLKO.1 shRNA targeting sequence (DHODH)	Sigma Aldrich	TRCN0000294663
CCGGTGGGCTGCCTCTGGGAATAAACTCGAGTTT ATTCCCAGAGGCAGCCCATTTTTG		
pLKO.1 shRNA targeting sequence (DHODH)	Sigma Aldrich	TRCN0000294666
CCGGTGAGCTGGAGGCCCTTCTAAACTCGAGTTT AGAAGGGCCTCCAGCTCATTTTTG		
pLKO.1 shRNA targeting sequence (DHODH)	Sigma Aldrich	TRCN0000287215
CCGGCGACCATTTCTACGCCGAGTACTCGAGTAC TCGGCGTAGAAATGGTCGTTTTTG		
pLKO.1 shRNA targeting sequence (DHODH)	Sigma Aldrich	TRCN0000294664
CCGGGAGGACCAAGCTGTTATTAACCTCGAGGTTA ATAACAGCTTGGTCCTCTTTTTG		
pLKO.1 shRNA targeting sequence (DHODH – chosen for this paper)	Sigma Aldrich	TRCN0000294665
CCGGCCACTGTCTCTAGATCTAAATCTCGAGATTTA GATCTAGAGACAGTGGTTTTTG		
pLL3.7shScrambled-hSyn-mCherry	Gazit et al., 2016	N/A
pLL3.7	Addgene	Addgene Plasmid #11795

**Biological Samples**		

GSE27268	Winden et al., 2011	https://www.ncbi.nlm.nih.gov/geo/
GSE14763	Okamoto et al., 2010	https://www.ncbi.nlm.nih.gov/geo/
GSE1155	Bough et al., 2006	https://www.ncbi.nlm.nih.gov/geo/
GSE112627	Hawkins et al., 2019	https://www.ncbi.nlm.nih.gov/geo/
E-MTAB-3123	N/A	https://www.ebi.ac.uk/arrayexpress/

**Chemicals, Peptides, and Recombinant Proteins**		

Teriflunomide	Tocris Bioscience	Cat# 5069; CAS: 108605-62-5
R-Baclofen	Tocris Bioscience	Cat# 0796; CAS: 69308-37-8
DL-TBOA	Tocris Bioscience	Cat# 1223; CAS: 205309-81-5
Uridine 5′-triphosphate trisodium salt hydrate	Sigma Aldrich	Cat# U6625; CAS: 19817-92-6
Tetrodotoxin	Alomone labs	Cat# T-550; CAS:18660-81-6
AP-5	Abcam	Cat# ab120271; CAS: 1303993-72-7
CNQX	Tocris Bioscience	Cat# 1045; CAS: 479347-85-8
Kynurenic acid	Tocris Bioscience	Cat# 0223; CAS: 492-27-3
Gabazine	Abcam	Cat# ab120042; CAS: 104104-50-9
PTZ	Sigma Aldrich	Cat#P6500; CAS:54-95-5
Oregon Green 488 BAPTA-1 AM (OGB-1 AM)	Invitrogen	Cat# O6807
Oligomycin	Sigma Aldrich	Cat# O4876
Antimycin A	Sigma Aldrich	Cat# A8674
Rotenone	Sigma Aldrich	Cat# R8875
Bam15	Tocris Bioscience	Cat# 5737
FCCP	Sigma Aldrich	Cat# C2920
2-Deoxy-D-glucose (2DG)	Sigma Aldrich	Cat# D6134

**Critical Commercial Assays**		

Seahorse XF96 V3 PET Culture Microplates	Agilent Technology	Cat#101104-004
Seahorse XFe96 FluxPak	Agilent Technology	Cat#102416-100
MasterPure DNA Purification kit	Epicenter	Cat #MCD85201
Fast SYBR Green Master Mix	Termo Fisher Scientific	Cat #4385612

**Experimental Models: Organisms/Strains**		

BALB/cOlaHsd	Envigo, Israel	stock # 162
Mouse: floxed stop Scn1a^∗^A1783V	The Jackson Laboratory	IMSR Cat# JAX:026133; RRID: IMSR_JAX:026133
Mouse: CMV-Cre	The Jackson Laboratory	IMSR Cat# JAX:006054; RRID: IMSR_JAX:006054

**Recombinant DNA**		

pCAG mito-RCaMP1h	Hirabayashi et al., 2017	Addgene Plasmid #105013

**Oligonucleotides**		

2mt-mCherry FORW	This paper	N/A
5′gagagcgcagtcgaattgctagcGCCACCATGGGCGGTAG GCGTGTACGGT		
2mt-mCherry REV	This paper	N/A
5′gatccaagcttgatatcactagtgaattcTTACTTGTACAGCTC GTCCATGCCGCCGGT		
Dloop1 FORW	West et al., 2015	N/A
5′AATCTACCATCCTCCGTGAAACC3′		
Dloop1 REV	West et al., 2015	N/A
5′TCAGTTTAGCTACCCCCAAGTTTAA3′		
TERT FORW	West et al., 2015	N/A
5′CTAGCTCATGTGTCAAGACCCTC3′		
TERT R 5′GCCAGCACGTTTCTCTGTT3′	West et al., 2015	N/A
HPRT FORW	Ruggiero et al., 2017	N/A
5′GCAGTACAGCCCCAAAATGG3′		
HPRT REV	Ruggiero et al., 2017	N/A
5′GGTCCTTTTCACCAGCAAGCT3′		
sh665 FORW	This paper	N/A
5′CCGGCCACTGTCTCTAGATCTAAATCTCGAGATTTA GATCTAGAGACAGTGGTTTTTG		
sh665 REV	This paper	N/A
5′CAAAAACCACTGTCTCTAGATCTAAATCTCGAGATTT AGATCTAGAGACAGTGGCCGG		
**Software and Algorithms**		

Wave Desktop 2.6	Agilent	https://www.agilent.com/en/products/cell-analysis/wave-controller-for-the-seahorse-xfe-analyzer
MATLAB	MathWorks	RRID: SCR_001622
Graphpad Prism 6		RRID: SCR_002798
MiniAnalysis	Synaptosoft, Decatur, Georgia, USA	RRID: SCR_002184
ImageJ- Fiji		RRID: SCR_002285
iMAT	Shlomi et al., 2008	N/A
MTA	Yizhak et al., 2013	N/A

### Contact for Reagent and Resource Sharing

Further information and requests for resources and reagents should be directed to and will be fulfilled by the Lead Contact, Inna Slutsky (islutsky@tauex.tau.ac.il).

### Experimental Model and Subject Details

All animal experiments were approved by the Tel Aviv University Committee on Animal Care.

#### Primary cultures

Hippocampi were dissected from BALB/c pups (both sexes) at P0-2 in ice cold Leibovitz L-15 medium. Cells were washed 3 times with HBSS and incubated in digestion solution (137 mM NaCl, 5 mM KCl, 7 mM Na2HPO4, 25 mM HEPES, 2 mg/ml trypsin, 0.5 mg/ml DNase) for 10 min. Then washed once with HBSS supplemented with 20% FBS to inactivate the protease and once again with HBSS alone. Cells were then dissociated in HBSS supplemented with 13 mM MgSO_4_ and 0.5 mg/ml DNase by tituration with fire-polished pipettes. Following centrifugation at 1000 r*cf.* for 10 min at 4°C, supernatant was removed, cells were re-suspended with plating medium (MEM supplemented with 10% FBS, 32.7 mM glucose, 25 mg/ml insulin, 2 mM Glutamax, 0.1 mg/ml transferrin, 0.1% SM1) and then plated on matrigel-coated glass coverslips or MEA plates. One day later, 50% of the serum medium was replaced with feeding medium (MEM supplemented with 32.7 mM glucose, 2 mM Glutamax, 3 μM ARA-C, 0.1 mg/ml transferrin, 2% SM1). Half of the medium was replaced twice a week with a fresh feeding medium. The experiments were performed in cultures after 14 – 21 days *in-vitro* (DIV).

#### Animals

Acute hippocampal slices were prepared from 2-month-old BALB/c mice of both sexes. *In vivo* experiments were performed in 2-month-old male BALB/c and in 2-4 month-old male and female DS mice, generated by crossing the conditional Scn1a-A1783V (The Jackson Laboratory stock 026133) with CMV-Cre mice (The Jackson Laboratory stock 006054). All animals were kept in a normal light/dark cycle (12h/12h), 3 animals per cage with access to food and water *ad libitum*.

### Method Details

#### Genome-scale metabolic modeling analysis (GSMM)

A metabolic network consisting of *m* metabolites and *n* reactions can be represented by a stoichiometric matrix S [2766X3742], where the entry *Sij* represents the stoichiometric coefficient of metabolite *i* in reaction *j*. Constraint-based modeling (CBM) imposes mass balance, directionality and flux capacity constraints on the space of possible fluxes in the metabolic network’s reactions through a set of linear equations

(1) S∗V=0

(2) Vmin<V<Vmax

V is a vector whose values describe the flux in each of the reactions in the model. The exchange of metabolites with the environment is represented as a set of transport reactions, enabling a predefined set of metabolites to be either taken up or secreted from the tissue. The steady-state assumption represented in Equation 1 constrains the production rate of each metabolite to be equal to its consumption rate. Enzymatic directionality and flux capacity constraints define lower and upper bounds on the fluxes and are embedded in Equation 2. In the following, flux vectors satisfying these conditions will be referred to as feasible steady-state flux distributions.

#### Our GSMM analysis proceeds along three major steps

(1) Based on CBM constraints arising from the human metabolic model and information derived from gene-expression data, the iMAT algorithm generates a feasible solution space of metabolic reactions activity that best coincides with the input expression data, thus simulating the post-transcriptional metabolic flux distribution in a given state. A first pre-requisite for using iMAT (Integrative Metabolic Analysis Tool) (Zur et al., 2010) is mapping the gene expression data into the human metabolic model. Genes were mapped according to their gene symbol to the model and later on discretized to highly, lowly or moderately expressed for each sample. This discretization was based on the ranking of the expression so that the top 25% were designated highly expressed while the bottom 25% as lowly expressed, the rest were designated moderately expressed. As each iMAT analysis requires a single vector of expression in each sample, only genes that were consistently highly or lowly expressed in 2/3 of its samples were defined so in the final input of the iMAT algorithm.
(2) Given these expression-derived constraints, the iMAT analysis computes the feasible flux space across the network using a mixed integer linear programming (MILP) approach designed to find a steady-state flux distribution satisfying stoichiometric and thermodynamic constraints, while maximizing the number of reactions whose activity is consistent with their expression (Shlomi et al., 2008). In order to find an optimized solution for the MILP problems presented we used the IBM CPLEX linear optimization tool. A total of 2000 solutions were sampled for each sample using Artificially Centered hit–and–run (ACHR) sampling supplied by Cobra toolbox 2.0 (Schellenberger et al., 2011) according to the solution space derived from iMAT. The mean value of each reaction flux across these solutions is then used to depict a sample’s metabolic state.
(3) This flux distribution output was then used as input for the MTA algorithm (Yizhak et al., 2013), which was utilized to search for drug targets that are likely to transform a metabolically compromised seizure-associated condition toward a healthy or neuroprotective protective one. MTA gets as input gene expression levels of two metabolic states, termed source and targets states. Next, the MTA approach works to (a) infer the most likely distribution of fluxes in the source state using iMAT; (b) identify the set of genes that their expression has significantly changed between the source and targets states, and the set of genes that their expression remains constant. Following, the algorithm searches for perturbations that can globally shift all the fluxes of the changed reactions in the desired direction, while keeping the fluxes of the unchanged reaction as close as possible to their predicted source state. Finally, MTA outputs a ranked list of candidate perturbations according to their ability to induce the desired transformation, from the source to the target metabolic state (Yizhak et al., 2013). In the MTA analysis, all dead-end reactions as well as artificial ones (reaction not mapped to genes) were filtered out leaving a total of 1504 reactions. The list of most likely metabolic perturbations was selected using a dual criterion: (1) The reactions should have a score higher than what would have been achieved without any perturbation; (2) In the top 10% scores in each prediction (150 reactions).
(4) MTA overall predictions were initially validated on a large scale by compiling a Seizure Predisposing Gene List: The list of seizure predisposing genes was derived using the MGI Phenotype disease and alleles query (Search Category: Seizure; MP: 0002064). First, a total of 327 genes were associated with knockout or overexpression experiments leading to increased seizure activity. Second, 38 Knockout metabolic genes were filtered in order to validate Epileptic MTA prediction sets. To define if these reactions were significantly enriched in MTA predictions we used a hypergeometric statistical test (see Table S2).

#### Plasmids, transfection, infection

For shRNA-mediated knockdown of DHODH, we chose the sequence of shRNA from Sigma Aldrich (TRCN0000294**665**, see Key Resources Table).

AAV-hSynI-2mtGCaMP6m was prepared by cloning 2mtGCaMP6m (Dr. Diego De Stefani, University of Padova) between BamHI and NotI sites of AAV2-hSynI (Dr. Daniel Gitler, Ben Gurion University).

AAV-hSynI-2mt-mCherry was constructed by Gibson assembly. Insert 2mt-mCherry was amplified by PCR with the following primers: forward 5′ gagagcgcagtcgaattgctagcGCCACCATGGGCGGTAGGCGTGTACGGT and reverse 5′ gatccaagcttgatatcactagtgaattcTTACTTGTACAGCTCGTCCATGCCGCCGGT, and assembled with AAV2-hSynI, pre-cut by NheI and EcoRI.

AAV-hSynI-jRGECO1a was prepared by cloning jRGECO1a (pGP-CMV-NES-jRGECO1a, Addgene plasmid # 61563) between BglII and NotI sites of AAV2-hSynI.

#### The integrity of the constructs was confirmed by the sequencing analysis

To quantify the knockdown efficiency, primary neuronal mice culture was infected with lentiviruses containing medium. The equal amount of cells was infected on DIV3 and collected for RNA extraction on DIV17. RNA was immediately extracted using the RNeasy Mini kit (QIAGEN Inc.) following the manufacturer’s protocol. The equal amount of mRNA was reverse-transcribed to cDNA with Superscript III reverse transcriptase (Invitrogen, cat. No:18080-051). Real-time qPCR was performed with TaqMan probes (Applied Biosystems) for DHODH (Mm00498393_m1) and GAPDH (Gapdh Mm99999915_g1), the latter served as an endogenous reference. Reactions were run in triplicate in a StepOnePlus real-time PCR system (Applied Biosystems). mRNA abundance was calculated by means of the comparative cycle threshold (Ct) method following the manufacturer’s guidelines. DHODH expression levels were reported as normalized to *GAPDH*.

For shDHODH experiments, infection was done at DIV 2-4 and experiments were performed at DIV 15-28.

#### Electrophysiology in hippocampal cultures and slices

##### MEA

Cultures were plated on MEA plates containing 120 titanium nitride (TiN) electrodes, in addition to 4 internal reference and 4 ground electrodes. Each electrode has a diameter of 30 μm and electrodes are arranged in a 12X12 grid (sparing 6 electrodes in each corner), spaced 100-200 μm apart on average [Multi Channel Systems (MCS), 120MEA200/30iR-Ti]. Data acquisition was done using a standard MEA2100-System (MCS) with a hardware filter cut-off of 3.3 kHz and sampling rate of 10 kHz per electrode. Recordings were carried out under constant 37°C and 5% CO2 levels similar to incubator conditions.

##### Data analysis

Raw data were filtered, offline, at 200 Hz using a Butterworth high-pass filter. Spikes were then detected, offline, using MC Rack software (MCS) based on a fixed threshold set to between 5-6 standard deviations from mean. Twenty minutes of each hour (that were previously shown to reliably represent the MFR of the entire hour, were used for analysis to reduce processing time and analyzed using custom-written scripts in MATLAB (Mathworks) as previously described (Slomowitz et al., 2015). Channels with unstable (> 30% change of MFR) baseline recordings during 3-4 h prior to a perturbation were excluded from the analysis. While data collection was not performed blind to the condition of experiment, investigator was blind to these conditions throughout much of the analysis, as spikes were automatically detected and the manual inspection of spikes happened without any knowledge of experimental conditions.

##### Patch clamp electrophysiology in hippocampal cultures

Experiments were performed at room temperature in a recording chamber on the stage of FV300 inverted confocal microscope (Olympus, Japan). Extracellular Tyrode solution contained (in mM): NaCl, 145; KCl, 3; glucose, 15; HEPES, 10; MgCl2, 1.2; CaCl2, 1.2; pH adjusted to 7.4 with NaOH. Whole-cell patch clamp internal solution for intrinsic excitability measurements contained (in mM): K-gluconate 120; KCl 10; HEPESs 10; Na-phosphocreatine 10; ATP-Na2 4; GTP-Na 0.3; MgCl2 0.5. In these recordings synaptic blockers (in μM, 25 DNQX, 50 AP-5, and 10 gabazine) were added to the Tyrode solution. For mEPSCs recordings, internal solution contained (in mM): Cs-MeSO3 102, CsCl 3.5, HEPES 10, Na2Phosphocreatine 8, CaCl2 1, Mg-ATP 4, Na-GTP 0.3, Cs-BAPTA 10, MgCl2 0.5, EGTA 0.5, QXCl 2. In these recordings tetrodotoxin (1 μM), AP-5 (50 μM), and gabazine (30 μM) were added to the Tyrode solution. For intrinsic excitability, frequency was measured by calculating the rate of action potentials in current-clamp during 500-ms long depolarizing steps of increasing intensity; a small DC current was injected to maintain membrane potential at −65 mV in between depolarizations. Input resistance (Rin) was measured by calculating the slope of the voltage change in response to increasing current injections. Neurons were excluded from the analysis if serial resistance was > 20 MΩ, and Rin was < 80 MΩ. Signals were recorded using MultiClamp 700B amplifier, digitized by DigiData1440A (Molecular Devices, Sunnyvale, California, USA) at 10 kHz, and filtered at 2 kHz. Electrophysiological data were analyzed using pClamp (Molecular Devices) and MiniAnalysis (Synaptosoft, Decatur, Georgia, USA) for mEPSC. The analysis was not blind to the experimental conditions.

##### Electrophysiology in slices

Acute hippocampal slices (coronal, 400 μm) were prepared from 2-month-old BALB/c as described before (Abramov et al., 2009). Slices were transferred to a submerged recovery chamber at 32°C containing oxygenated (95% O2 and 5% CO2) artificial cerebrospinal fluid (ACSF) for 1h before the experiment. The ACSF contained, in mM: NaCl, 125; KCl, 2.5; CaCl2, 1.2; MgCl2, 1.2; NaHCO3, 25; NaH2PO4, 1.25; glucose, 25. fEPSPs were recorded in acute hippocampal slices with a glass pipette containing Tyrode solution (1 – 2 MΩ) from synapses in the CA1 stratum radiatum using a MultiClamp700B amplifier (Molecular Devices). Stimulation of the Shaffer Collateral (SC) pathway was delivered through a glass suction electrode (10 – 20 μm tip) filled with Tyrode. Data were analyzed using pClamp10 (Molecular Devices).

For mEPSC recording in slices, 1 μM TTX was added to ASCF to block spiking activity and slices were incubated with 50 μM TERI or the same concertation of VEH for 30 min prior to patch. CA1 pyramidal cells were patched using glass pipette (3-4 MΩ) containing the following intracellular solution (in mM): Cs-MeSO3 102, CsCl 3.5, HEPES 10, Na2Phosphocreatine 8, CaCl2 1, Mg-ATP 4, Na-GTP 0.3, Cs-BAPTA 10, MgCl2 0.5, EGTA 0.5. Cells were held at −70 mV during recording. Events were analyzed using MiniAnalysis (Synaptosoft, Decatur, Georgia, USA).

The analysis was not blind to the experimental conditions.

#### Confocal imaging in hippocampal cultures

Hippocampal neurons were imaged using a FV1000 spectral Olympus confocal microscope using a 60 × 1.2 NA water-immersion objective.

##### Synaptic ATP measurements

Images were 512 × 512 pixels, with a pixel width of 92 – 110 nm. Experiments were conducted at room temperature in Tyrode solution. Intensity-based FRET imaging was carried as described before (Gazit et al., 2016). Briefly, in neurons expressing SypI-ATeam1.03 (Shulman et al., 2015), the donor (mseCFP) was excited at 440 nm, and its emission was measured at 460-500 nm before (*I*_DA_) and after (*I*_D_) acceptor (cpmVen) photobleaching. Excitation was delivered to the acceptor at 514 nm, and emission was measured at 530–600 nm. Photobleaching of cpmVen was carried out with the 514 nm laser line, by a single-point activation module for rapid and efficient multi-region bleaching. The FRET efficiency, *E*_m_, was calculated as *E*_m_ = (*I*_DA_)/*I*_D_. Synapses that showed less than 85% reduction in donor signal after bleaching were excluded. Time-course measurements of FRET ratio were carried as described before (Shulman et al., 2015). Briefly, SypI-ATeam1.03 expressing synapses were excited at 440 nm and emission for mesCFP (460-500 nm, *I*_donor_) and cpmVen (530-600 nm, *I*_acceptor_) was measured over 5 min intervals. As 40% of I_acceptor_ originates from mseCFP, we used a correction for the tail of mseCFP’s emission, and FRET ratio was calculated as follows:

$$\text{FRET}\;\text{ratio}=\left(I_{\text{acceptor}}–0.4*I_{\text{donor}}\right)/I_{\text{donor}}$$

The cmpVen stability was measured by excitation at 514 nm to exclude changes of focus and bleaching during the time of the experiment. Synapses displaying > 15% changes in cmpVen emission were excluded from analysis.

#### Calcium imaging

For monitoring presynaptic cytosolic Ca^2+^ transients and resting cytosolic Ca^2+^, fluorescent calcium indicator Oregon Green 488 BAPTA-1 AM (OGB-1 AM) was dissolved in DMSO to yield a concentration of 1 mM. For cell loading, cultures were incubated at 37°C for 30 min with 3 μM of this solution diluted in Tyrode solution (Gazit et al., 2016). Activity-dependent FM4-64 styryl dyes were used to label functional presynaptic terminals by 600APs @ 20Hz. 500 Hz line scanning during stimulation was used to specifically detect Ca^2+^ transients of OGB-1 AM at FM-(+) synapses. For resting cytosolic Ca^2+^ measurements, TTX (1 μM) was added to the Tyrode solution to block Ca^2+^ changes resulting from spiking activity. For detection of mitochondrial Ca^2+^ (mito-Ca^2+^), neurons were co-transfected with 2mtGCaMP6m and mCherry-mito. Resting mito-Ca^2+^ was measured in the presence of 1 μM TTX. Mitochondrial Ca^2+^ evoked by spikes (5 APs @ 50 Hz) was measured in the presence of 2.5 μM DNQX to block recurrent activity. Only axonal mitochondria were analyzed. Imaging was performed using FV1000 Olympus confocal microscope under 488 nm (excitation) and 510 – 570 nm (emission) for OGB1-AM and 2mtGCaMP6m, and 561 nm (excitation) and 575 – 675 nm (emission) for mCherry-mito. For mitochondrial Ca^2+^ measurements, 30 ROIs were manually selected per cell using co-localization of mCherry-mito and 2mtGCaMP6m. Data analysis was preformed using ImageJ software.

For simultaneous monitoring of somatic cyto-Ca^2+^ and mito-Ca^2+^, neurons were infected with both, jRGECO1a and 2mtGCaMP6m, excited by 488 and 561 nm laser, respectively. 1-2 min recordings (at 5 Hz frame rate) of emission from 2 channels ([505 – 540] and [575 – 675] nm emission) were taken simultaneously. Activity was tracked in the microscope stage incubator chamber (37°C, 5% CO2) during baseline, and 5, 15 and 30 min after the addition of PTZ (10 mM).

Analysis: Activity in mitochondria and cytosolic ROIs was quantified from the AUC of ΔF/F’ using ImageJ-Fiji software and custom routines in MATLAB (Mathworks). To account for changes in the baseline levels of Ca^2+^, AUC of ΔF/F_Bsl_ of the recorded trace was calculated, whereas F_Bsl_ corresponds to the baseline activity before addition of PTZ.

#### Respiration measurements

For the respiration studies, primary hippocampal neurons were plated on Matrigel pre-coated XF 96 plates (Agilent Seahorse Bioscience, Cat. # 101104-004) at a density of 30 × 10^5^ cells/well and cultured for 15 days. On the day of the experiment, cells were washed three times and pre-incubated for 1 h in Assay Media (bicarbonate-free unbuffered DMEM medium; Sigma, Cat. # D5030) supplemented with 31.6mM NaCl, 10mM Glucose, 2mM Na-Pyruvate, 2mM Glutamax and NeuroCult SM1. Measurement of intact cellular respiration was performed using the Seahorse XF96 analyzer (Agilent) and the XF Cell Mito Stress Test Kit according to the manufacturer’s instructions and as described (Llorente-Folch et al., 2013, Ruggiero et al., 2017). Respiration was measured under basal conditions, and in response to 1 μM Oligomycin (Sigma, Cat. # O4876) followed by the addition of the electron transport chain accelerator ionophore 4-(trifluoromethoxy) phenylhydrazone (FCCP; 3 μM. Sigma, Cat. # C2920) which induces maximal OCR (Oxygen Consumption Rate). Finally, respiration was stopped by adding the electron transport chain inhibitors Rotenone and Antimycin A (1 μM and 2 μg/mL respectively; Sigma, Cat. #R8875 and #1397-94-0). In other set of experiments (Figures 2D and 2E), Oligomycin was omitted to correct for its possible effects on the estimation of the maximal uncoupled respiration (Brand and Nicholls, 2011). Values were normalized to cellular protein levels. Coupling efficiency was calculated as ATP-linked OCR / basal OCR. Non-mitochondrial oxygen consumption was calculated as the minimum rate measurement after AA/Rot injection.

#### Mitochondrial DNA quantification

Total DNA was isolated from DIV15 primary hippocampal neurons using MasterPure DNA Purification kit (Cat #MCD85201, Epicenter). Samples were then sonicated for 5 min, and quantitative real-time PCR was performed in the presence of SYBR Green (Cat #4385612, Applied Biosystems). Expression levels were determined using the comparative cycle threshold (2-ΔΔCt) method, and hypoxanthine guanine phosphoribosyl transferase (HPRT) served as a housekeeping gene. Primer sequences used for the genes tested are listed below:

Dloop1 F 5′-AATCTACCATCCTCCGTGAAACC-3′
Dloop1 R 5′-TCAGTTTAGCTACCCCCAAGTTTAA-3′
TERT F 5′- CTAGCTCATGTGTCAAGACCCTC −3′
TERT R 5′- GCCAGCACGTTTCTCTGTT −3′
HPRT F-5′-GCAGTACAGCCCCAAAATGG-3′
HPRT R-5′-GGTCCTTTTCACCAGCAAGCT-3′

#### Metabolic profiling

Intracellular metabolites were extracted from hippocampal cultures with a solution of methanol: acetonitrile: water (5:3:2 ratio) on top of an ethanol / dry ice bath. Samples were rotated at 4°C for 10 min, cleared by centrifugation at 14,000 x g for 10 min, and stored at −80°C. LC-MS/MS analyses were performed on an Ultimate3000 UHPLC system (Dionex, Thermo Scientific) coupled to a Q-Exactive Plus mass spectrometer (Thermo Scientific). Metabolite separation was performed using SeQuant ZIC-pHILIC column (Merck; 150 × 2.1 mm, 5 μm) coupled to a SeQuant ZIC-pHILIC guard column (Merck; 20 × 2.1 mm, 5 μm) with flow rate of 0.1 ml/min. Metabolites were separated with a 49 min gradient of buffer A (95% acetonitrile) and buffer B (50 mM ammonium carbonate, pH 10,5% acetonitrile). Data were acquired using full MS scans and by switching between negative and positive polarity modes. Identification of metabolites of interest was done using LCquan software (Thermo Scientific) based on external standards, with mass tolerance of 3 ppm for uridine and 15 ppm for DHO

#### Surgical procedure for ICV injections

Mice were surgically prepared for intracerebroventricular (i.c.v.) injections. Briefly, the mice were anaesthetized with an intraperitoneal (i.p.) injection of ketamine/xylazine (80mg/kg ketamine and 15mg/kg xylazine), head fixed to a stereotaxic apparatus (David Kopf instruments) and maintained anesthetized by continuous isoflurane (1.5%) inhalation. Eye ointment was used to protect the mice eyes (Duratears, Vetmarket) and deep body temperature was recorded and maintained by a heating pad (FHC, DC temperature controller) at 36°C throughout the surgery. A small hole was drilled in the skull above the left lateral ventricle (0.7mm posterior, 1.2mm lateral to bregma), and a 5mm guide cannula was slowly inserted into the ventricle and fixed to the skull by dental cement (C&B Metabond, Parkell). The guide cannula was sealed with a 5mm sterile metal bar to prevent CSF leakage and possible infections. 1-2 weeks after the surgery, the mice received i.c.v. injections using a 10 μl syringe (Hamilton company) once a day, for three consecutive days. The mice were injected with 1 μl containing 27 μg of Teriflunomide (TERI), dissolved in DMSO or vehicle (VEH, 1 μl of DMSO) in speed of 0.15 μl/min (Nano Jet stereotaxic syringe pump). In order to confirm the accurate placement of the guide cannula into the lateral ventricle, at least 5 mice from each group (TERI / VEH) were submitted to dye injection (Trypan blue dye solution) in the end of experiments.

#### Electrophysiology *in vivo*

Mice were randomly assigned to the experimental groups. The animals were anaesthetized with an intraperitoneal (i.p.) injection of ketamine/xylazine (80 mg/kg ketamine and 15 mg/kg xylazine), placed in a stereotaxic frame for recordings and maintained under anesthesia by continuous isoflurane (1.5%) inhalation. Eye ointment was used to protect the mice eyes and deep body temperature was recorded and maintained by a heating pad at 36°C throughout the surgery.

Data collection was not performed blind to the condition of experiment. Investigators were blind to these conditions throughout much of the electrophysiological analysis (spikes were automatically detected and the manual inspection of spikes happened without any knowledge of the analyzed condition).

##### fEPSP/LFP recordings

Small holes were drilled in the skull at the position of the recording and stimulating electrodes, contralateral to the hemisphere of the guide cannula. The recording electrode (bipolar stainless steel; 0.127 mm diameter) was slowly lowered through the cortex into the CA1 *stratum radiatum* (2.06 mm posterior to bregma; 1.5 mm ML; 1.5 DV), and the stimulating electrode (bipolar stainless steel; 0.127 mm diameter) was slowly lowered through the cortex into the Schaffer-collateral (SC, 2.54 mm posterior to bregma; 2.75 mm ML; 2.2 mm DV). Ground electrode was screwed to the skull above the cerebellum. Test stimuli of 0.5mA were delivered to the SC at 0.06Hz to verify the proper location of the electrodes and to estimate the stability of the signal over 30 min prior to the start of recordings. Extracellular field potentials were amplified using a costume made amplifier x100, bandpass filtered between 0.1 Hz and 4 KHz, and digitized by Digidata 1440A (Molecular Devices). Data were analyzed using pCLAMP 10 (Molecular devices) for fEPSP’s. The measurements were 2-4 h after the third injection of TERI / VEH (Figures 6E–6H).

##### Single-unit recordings

Four to seven months old mice were implanted with a costume-made microdrive (custom printed circuit board and drive by Rogat, Carmiel, Israel) and a 5 mm cannula for TERI/VEH i.c.v. injection. The microdrive contained a moveable assembly of 4 tetrodes (17-μm, Platinum 10% Iridium, California Fine Wire) and was connected to the recording setup via an Omnetics headstage connector (Connector Corporation, Minneapolis MN, USA). Three holes were drilled in the skull: one in the frontal bone plate for a screw serving as ground; the second hole for the electrodes implanted in the parietal cortex (1.94 mm posterior of bregma; 1-1.2 mm medial lateral axis; 1-1.2 mm dorsal ventral axis), and the third hole - for the cannula implanted in the lateral ventricle ipsilateral to the recording site (0.46 mm posterior of bregma;1 mm ML; 2 mm DV). The cannula was sealed with a 5mm sterile metal bar to prevent CSF leakage and possible infections. After 7 days of monitored recovery, subsequent downward movements of the microdrive were made in 25- to 50-μm increments over 24-h intervals until approaching the CA1 pyramidal cell layer, recognized by the appearance of multiple high-amplitude units and spontaneous ripple events. At the end of the experiment, a small electrolytic lesion was made (30 μA for 20 s) under anesthesia. Two days after, histology procedure was performed to verify electrodes location as described (Weiss et al., 2017).

##### Single-unit data collection and analysis

Animals were placed in a familiar open field made of plexiglass (27 X 42 cm) and allowed to move freely. Each session consisted of 2 to 4 h of pre- and post-injection recordings. Raw data were sampled at 24 KHz using a Neurophysiology Workstation (RZ5D base processor and PZ5 NeuroDigitizer amplifier, Tucker-Davis Technologies Inc). Offline, spikes were extracted by passing the raw data through a median filter (window half-length = 10 samples) and setting a threshold of ± 2 MAD. Spike waveforms (32 samples symmetrical around the peak) were semi-automatically clustered using KlustaKwik (Kadir et al., 2014) followed by manual inspection using Klusters (Hazan et al., 2006). All subsequent data analysis was done using MATLAB (Mathworks, Natick MA). Clusters were defined as single units and included in the analysis only if they fulfilled the following criteria: (1) the presence of a refractory period (less than 1% of inter-spike intervals < 3 ms (Tankus et al., 2009); (2) an Isolation Distance > 20 (Harris et al., 2001, Harris et al., 2016), (3) a mean firing rate greater than 0.05 Hz during the baseline (pre-injection) period, and (4) a stable baseline firing rate during the pre-injection period.

#### PTZ seizure model

Pentylenetetrazole (PTZ, Sigma) was dissolved in PBS and administrated intraperitoneally at a dose of 70 mg/kg. Mice were randomly assigned to experimental groups. Each mouse was immediately and singularly placed in a 20 × 15 × 20 cm costume-made plexiglas observation box and observed for 30 min after PTZ administration by video recordings. Recordings were visually analyzed to quantify the severity score (Roberson et al., 2011), susceptibility score (Naydenov et al., 2014) and a revised Racine scale for PTZ (Lüttjohann et al., 2009). Behavior score was correlated to EEG-based score (Figure S12). All behavioral scoring was carried out blind to treatment.

#### Thermal induction of seizures

Mice were randomly assigned to experimental groups. The body core temperature of DS mice was monitored during the whole procedure, using a rectal temperature probe. The temperature was controlled with a feedback temperature controller and a heat lamp (TCAT2DF; Physitemp, Clifton, NJ). The mouse was allowed to acclimate to the chamber for 10 min and then its body temperature was elevated by 0.5°C every 2 min until a generalized tonic-clonic seizure (GTC) occurred or a body temperature of 42°C was reached. All behavioral scoring was carried out blind to genotype and treatment.

### Quantification and Statistical Analysis

Sample sizes were not statistically determined but were consistent with previous work using related methodology. The statistical tests used for the analysis of each type of experiments are specified in figure legends. Each experimental condition was replicated at least in 3 different mice / batches of cultures. Replication attempts were successful in independent samples. Statistical analysis was performed using Prism 6.0 GraphPad. The statistical test used, the consequent p value and the number of cells / mice that went into the calculation (n) are reported in the main text describing each figure. Data reported in the text are shown as mean ± standard error of the mean (SEM).

## Supplemental Information

Document S1. Figures S1–S12 and Tables S1, S2, and S4

Table S3. Selected Metabolic Perturbations Predicted to Reinstate a Healthy Metabolic State, Related to Figure 1

Document S2. Article plus Supplemental Information
