# Supplementary material for: Mitochondrial Regulation of the Hippocampal Firing Rate Set Point and Seizure Susceptibility
Source: Neuron. 2019 Jun 5;102(5):1009–1024.e8. doi: 10.1016/j.neuron.2019.03.045 (PMC6559804; doi:10.1016/j.neuron.2019.03.045)
Supplement: Document S1. Figures S1–S12 and Tables S1, S2, and S4 [file mmc1.pdf]

Neuron, Volume *102*

Supplemental Information

Mitochondrial Regulation of the Hippocampal

Firing Rate Set Point and Seizure Susceptibility

Boaz Styr, Nir Gonen, Daniel Zarhin, Antonella Ruggiero, Refaela Atsmon, Neta Gazit, Gabriella Braun, Samuel Frere, Irena Vertkin, Ilana Shapira, Michal Harel, Leore R. Heim, Maxim Katsenelson, Ohad Rechnitz, Saja Fadila, Dori Derdikman, Moran Rubinstein, Tamar Geiger, Eytan Ruppin, and Inna Slutsky

Supplementary Figures

Figure S1. Teriflunomide induces a reversible reduction in MFR, related to Figure 1

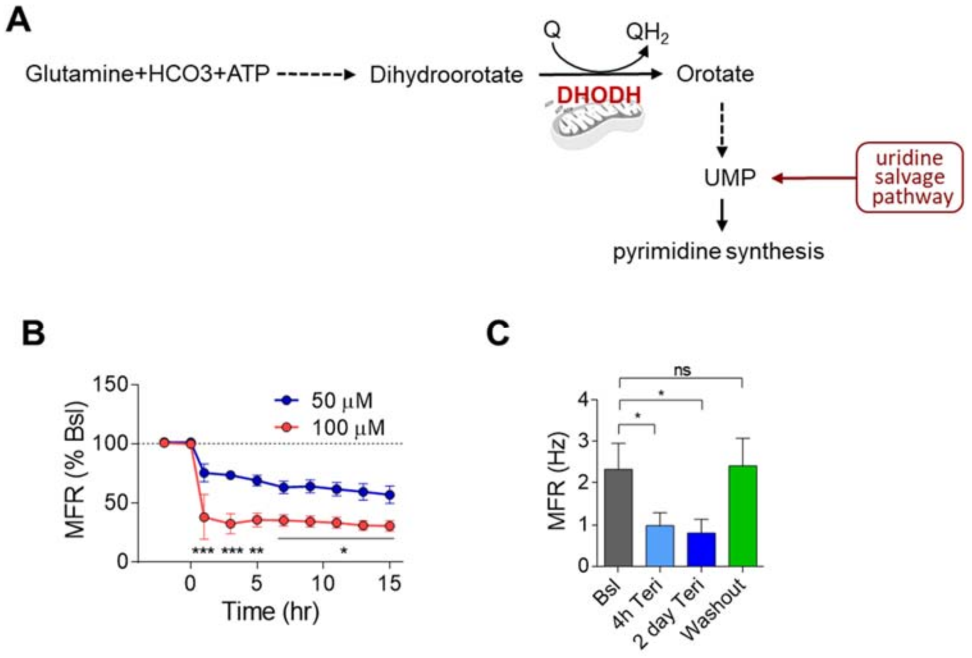

(A) The enzymatic step mediated by mitochondrial enzyme DHODH in *de novo* pyrimidine synthesis.

(B) Comparison of the effect of 100 μM (red, n = 6 experiments) and 50 μM (blue, n = 8 experiments) TERI on MFR using MEA recordings.

(C) Washout of TERI after 2 days restores original MFR (n = 17).

Two-way ANOVA with Sidak's multiple comparisons test (B), one-way ANOVA with Dunnett's multiple comparisons test (C). ***$p < 0.001$, **$p < 0.01$, *$p < 0.05$, ns – non-significant ($p > 0.05$). Error bars represent SEM.

Figure 2. Teriflunomide inhibits CA3-CA1 synaptic transmission and enhances synaptic facilitation in acute hippocampal slices. related to Figure 1

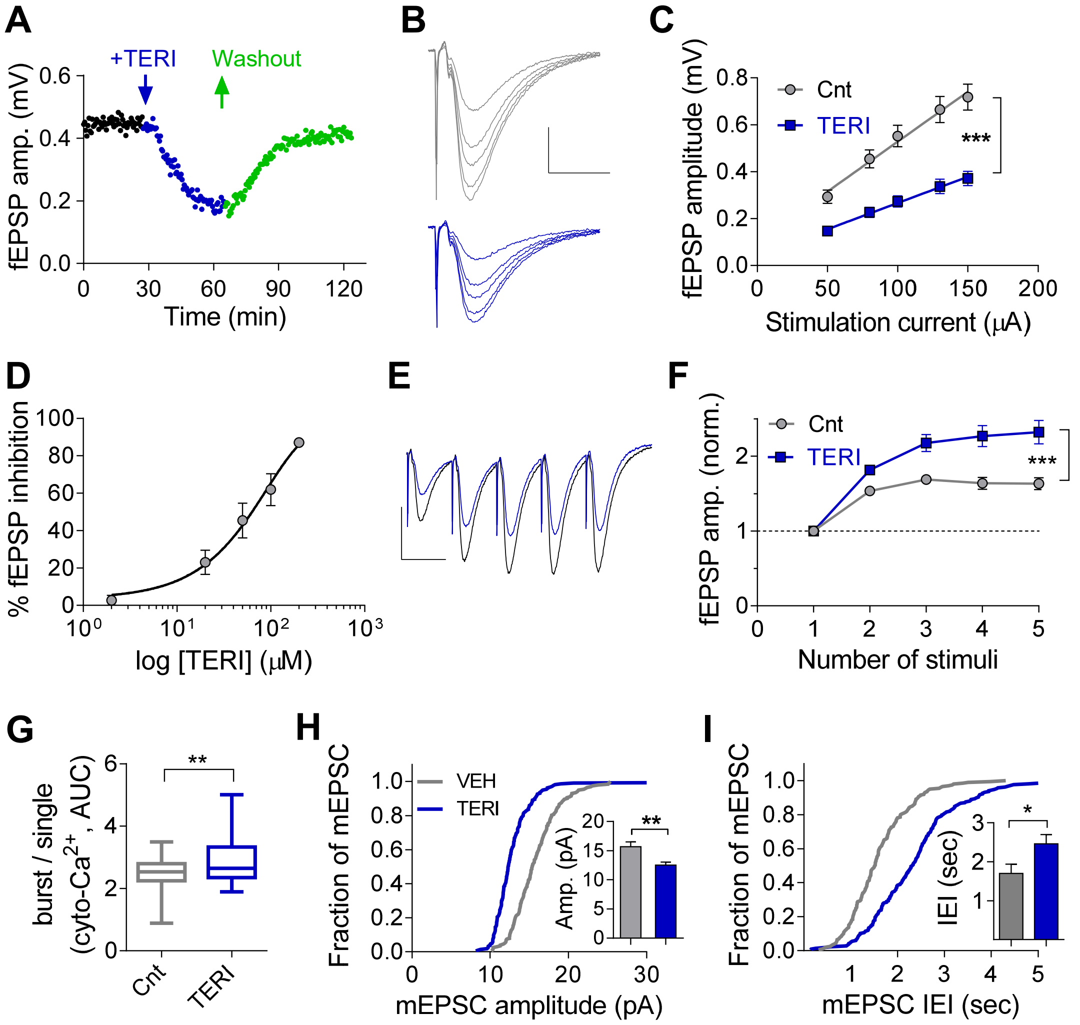

(A) Time-course of TERI (50 μM) and its washout on fEPSP amplitude.

(B) Representative fEPSP recordings before (black) and 30 min after (blue) application of TERI under low frequency stimulation (0.1 Hz). Scale bars: 0.3 mV, 10 ms.

(C) Effect of 50 μM TERI on input-output relationship between the intensity of stimulation and the fEPSP amplitude (n = 9 experiments, 5 mice).

(D) Dose response of TERI on fEPSP (n = 4 experiments, 4 mice).

(E) Representative recordings of fEPSP evoked by bursts (5 stimuli at 50 Hz) before (black) and 30 min after (blue) application of TERI. Scale bars: 0.5 mV, 20 ms.

(F) Relative effect of 50 µM TERI on peak amplitude of each fEPSP in the burst normalized to the first fEPSP amplitude (n = 9 experiments, 5 mice).

(G) TERI (50 µM) increases the facilitation of presynaptic cyto-Ca^{2+} during bursts (5 APs @ 50 Hz, n = 52 boutons).

(H) Cumulative histograms of mEPSC amplitudes in the CA1 pyramidal neurons after vehicle (VEH, n = 9 cells) or acute TERI application (n = 10 cells) in hippocampal slices. *Insert*: TERI reduced mean mEPSC amplitude from 15.7 pA to 12.5 pA.

(I) Cumulative histograms of mEPSC inter-event-intervals (IEIs) in the CA1 pyramidal neurons after vehicle (VEH, n = 9 cells) or acute TERI application (n = 10 cells) in hippocampal slices. *Insert*: TERI increased mean mEPSC IEI from 1.70 sec to 2.46 sec.

Two-way ANOVA with Sidak's multiple comparisons test (C,F), Unpaired, two-tailed student t-test (G-I). ***$p < 0.001$, **$p < 0.01$, *$p < 0.05$. Error bars represent SEM.

Figure S3. DHODH knockdown by shRNA-mediated approach, related to Figure 1

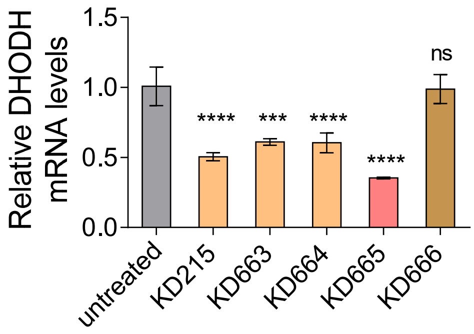

Knockdown efficiency of different shRNA sequences tested (see methods). Sequence KD665 was selected as the most potent and used for functional experiments (denoted as shDHODH in the main text). One-way ANOVA with Dunnett's multiple comparisons test.

****p < 0.0001, ***p < 0.001, ns – non-significant (p > 0.05). Error bars represent SEM.

Figure S4. Mitochondrial inhibition occludes the effect of teriflunomide on synaptic and neuronal activity, related to Figure 2

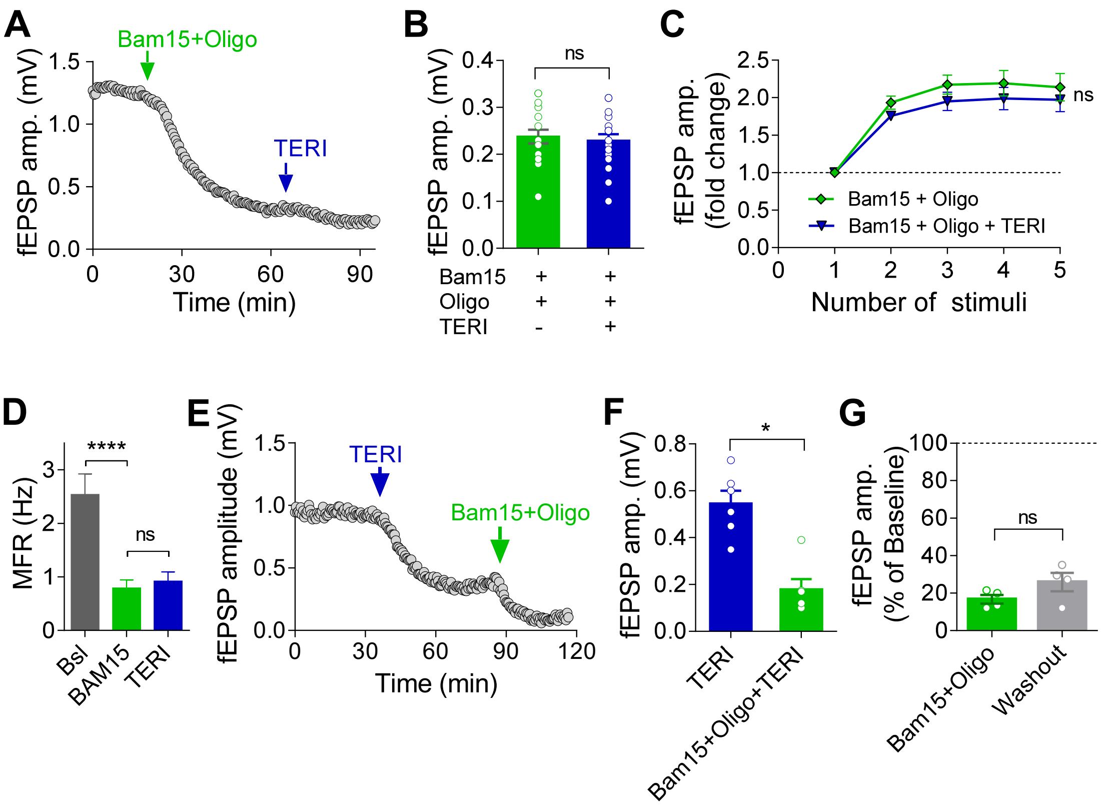

(A-B) Bam15 (10 µM) + Oligomycin (Oligo, 1 µg/ml) occluded the effect of TERI (50 µM) on fEPSP amplitude (n = 17 experiments, p = 0.49).

(C) Mitochondrial blockers (Bam15 + Oligo) occluded the effect of 50 µM TERI on short-term synaptic facilitation (n = 9 experiments, p > 0.05).

(D) Bam15 (2 µM) inhibited MFR (*P* < 0.0001) and occluded the effect of TERI (50 µM, 6 hr, *P* > 0.05) in hippocampal cultures grown on MEA (45 channels).

(E-F) TERI (50 µM) did not occlude the effect of Bam15 + Oligo on fEPSP amplitude in hippocampal slices (n = 6 experiments, p = 0.03).

(G) Bam15+Oligo (10 µM + 1 µg/ml) produced irreversible inhibition of fEPSP amplitude (n = 4 experiments, p = 0.25).

Wilcoxon matched-pairs signed rank test (B,F,G), two-way ANOVA (C), one-way ANOVA with Tukey's multiple comparisons test (D). ****$p < 0.0001$, *$p < 0.05$, ns – non-significant ($p > 0.05$). Error bars represent SEM.

Figure S5. Effects of teriflunomide on mitochondrial functions, related to Figure 2

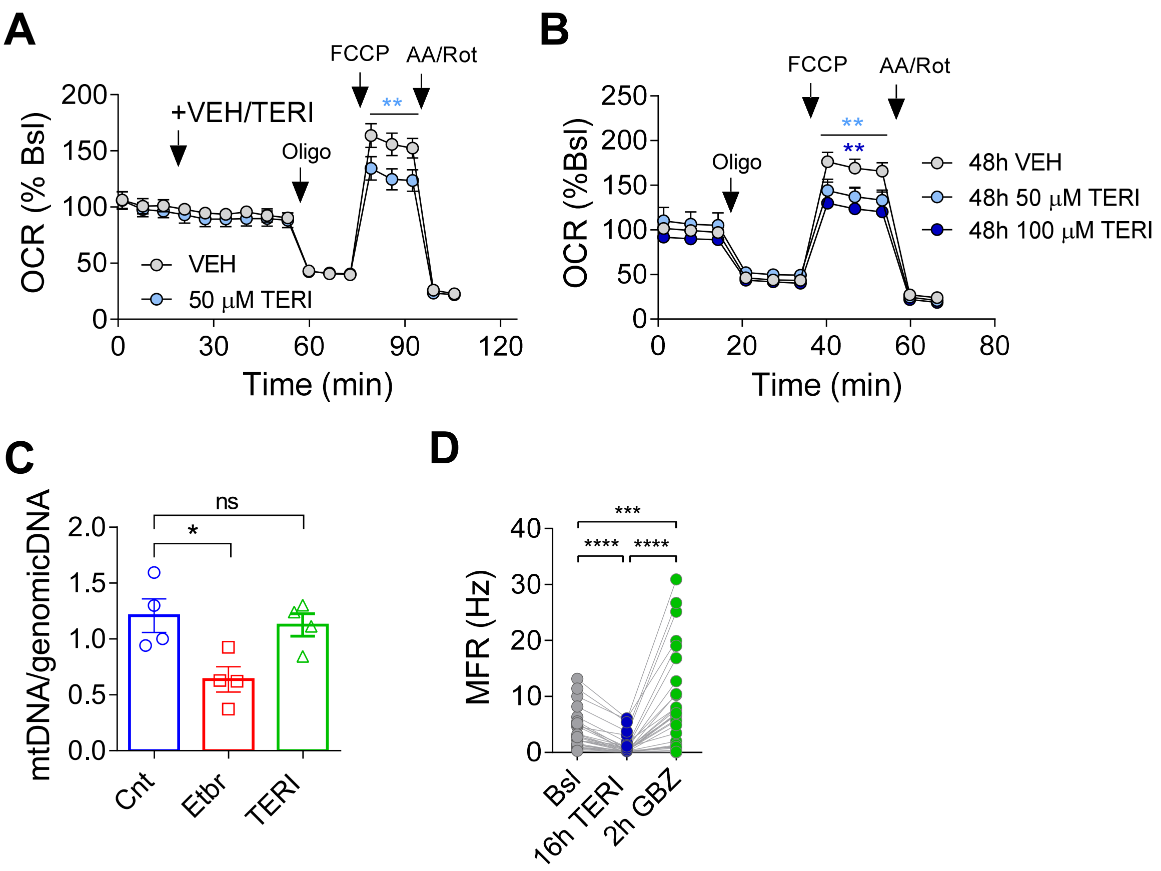

(A) Seahorse Bioscience XF96 analyzer was used to determine oxygen consumption rates (OCR) in intact DIV15 primary hippocampal neurons treated acutely with 50 μM TERI or VEH. OCR was measured under basal condition and after the addition of 1 μM Oligomycin (Oligo), 3 μM FCCP and 1 μM Rotenone (Rot) +2 μg/mL antimycin A (AA). Graph represents normalized average of 4-5 wells per condition.

(B) Oxygen consumption rates (OCR) in intact DIV15 primary hippocampal neurons treated for 48h with 50 μM or 100 μM TERI. Graph represents normalized average of 2 independent

experiments; 5 wells per condition. Normalization is performed to the baseline of VEH group. No difference was observed between 50 μM or 100 μM TERI.

(C) Chronic TERI treatment doesn't affect mitochondrial mass (n = 4 experiments, one-way ANOVA with Dunnett's multiple comparisons test). Mitochondrial mass was estimated by measuring the ratio between mitochondrial DNA to nuclear DNA. DNA was extracted from primary hippocampal neurons DIV15 treated for 48h with 100 μM TERI. Dloop1 expression was measured by RT-PCR relative to HPRT; TERT served as a control of a nuclear encoded gene. 100 ng/mL EtBr was used as positive control.

(D) Application of $GABA_A$ receptor blocker gabazine (GBZ, 30 μM) increased MFR of hippocampal neurons treated with 50 μM TERI for 16 hr (n = 31 channels).

Two-way ANOVA with Sidak's multiple comparisons test (A), Two-way ANOVA with Tukey's multiple comparisons test (B), one-way ANOVA with Dunnett's multiple comparisons test (C), one-way ANOVA with Tukey's multiple comparisons test (D).

*p < 0.05, **p < 0.01; ***p < 0.001, ****p < 0.0001, ns – non-significant (p > 0.05). Error bars represent SEM.

Figure S6. Teriflunomide increases resting cytosolic Ca^{2+}, while decreasing resting mitochondrial Ca^{2+}, related to Figure 3

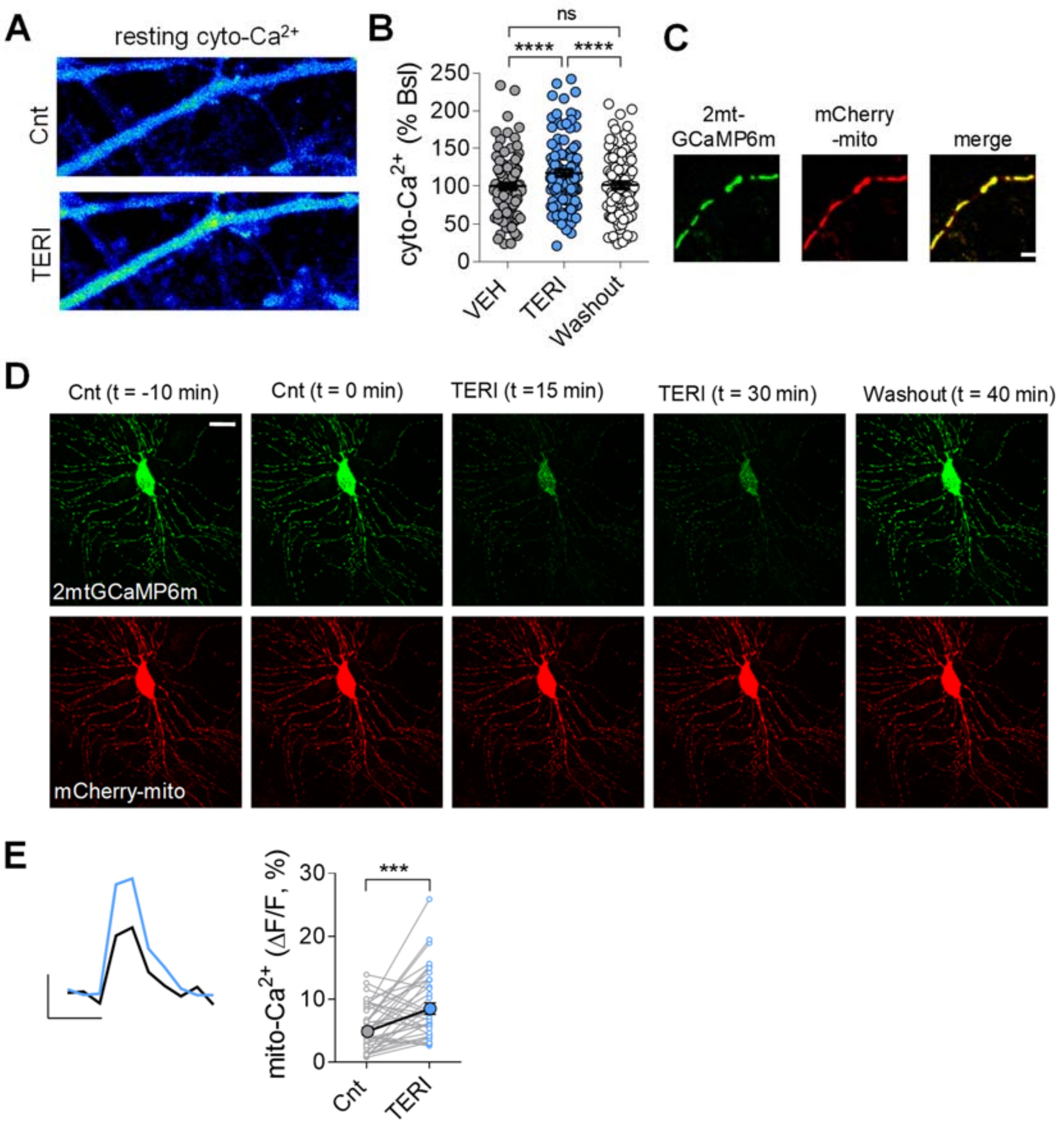

(A) Example of neuronal processes loaded with Oregon Green 488 BAPTA-1 AM before and after TERI application.

(B) TERI induced an elevation in resting cytosolic Ca^{2+} to 117 ± 4.6 % of the baseline which was reversible upon washout (n = 124).

(C) Colocalization of 2mtGCaMP6m with mCherry-mito in hippocampal processes. Scale bar: 5 μm.

(D) Representative images of a neuron co-transfected with mCherry-mito and 2mtGCaMP6m in baseline, during TERI application and after washout. Scale bar: 20 µm.

(E) *Left*: Average traces of mito-Ca^{2+} transients before (grey) and 30 min after (blue) evoked by 0.016 Hz stimulation and quantified as $\Delta F/F$. *Right*: TERI (50 µM, 30 min) increases mito-Ca^{2+} evoked by low-frequency, single AP stimulation (n = 36). Scale bars: 3% $\Delta F/F$, 1 sec.

One-way ANOVA with Tukey's multiple comparison test (B), Wilcoxon matched-pairs signed rank test (E). ****$p < 0.0001$, ***$p < 0.001$, ns – non significant ($p > 0.05$). Error bars represent SEM.

Figure S7. Regulation of intrinsic excitability by DHODH, related to Figure 4

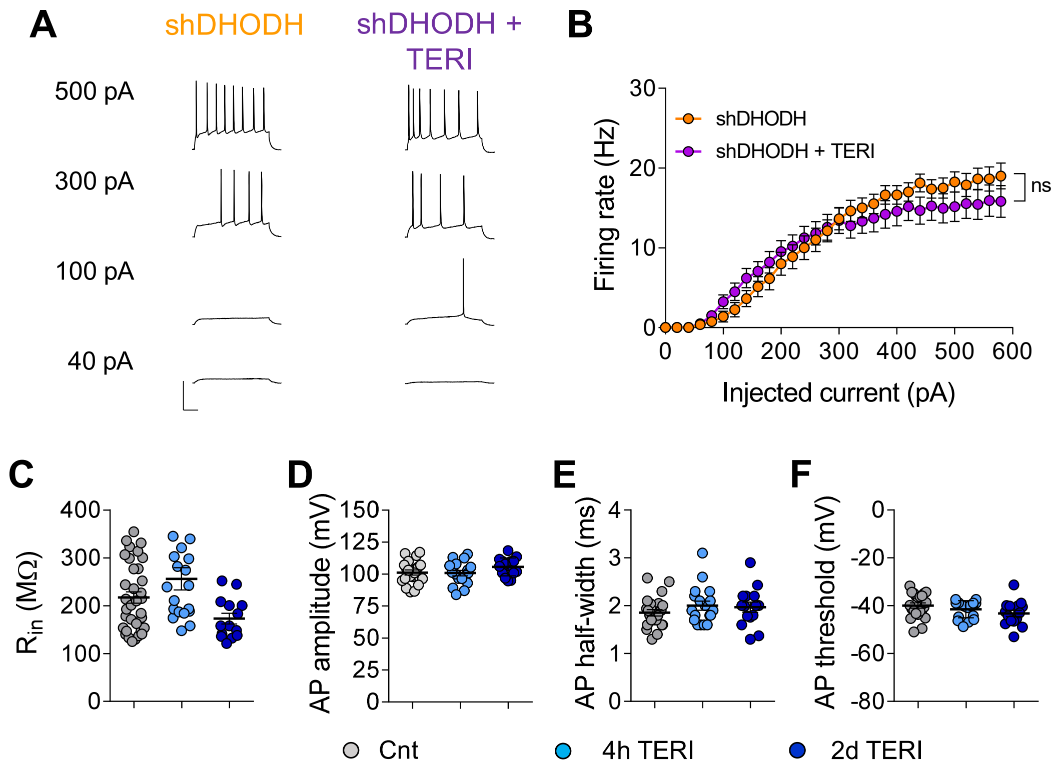

(A) Representative traces of voltage responses evoked by 20 pA step of current injections in shDHODH infected neurons with and without 4hr TERI (scale bars: 50 mV, 100 ms).

(B) F–I relationship. shDHODH occluded the effect of 4 hr TERI (shDHODH, n = 19, shDHODH + TERI, n = 21; $p > 0.05$).

(C-F) TERI did not affect neuronal input resistance (R_{in}) and single AP properties (n = 36, 19, 14 for control, 4 hr and 2 days TERI, respectively; $p > 0.05$). TERI did not alter input resistance (C), AP amplitude (D), half width (E) or AP threshold (F).

Two-way ANOVA with Tukey's multiple comparisons test (B), One-way ANOVA with Dunnett's multiple comparison test (C-F). ns – non-significant ($p > 0.05$). Error bars represent SEM.

Figure S8. Comparison of the effects of DHODH inhibition and partial mitochondrial uncoupling on MFR stabilization following chronic inactivity, related to Figure 5

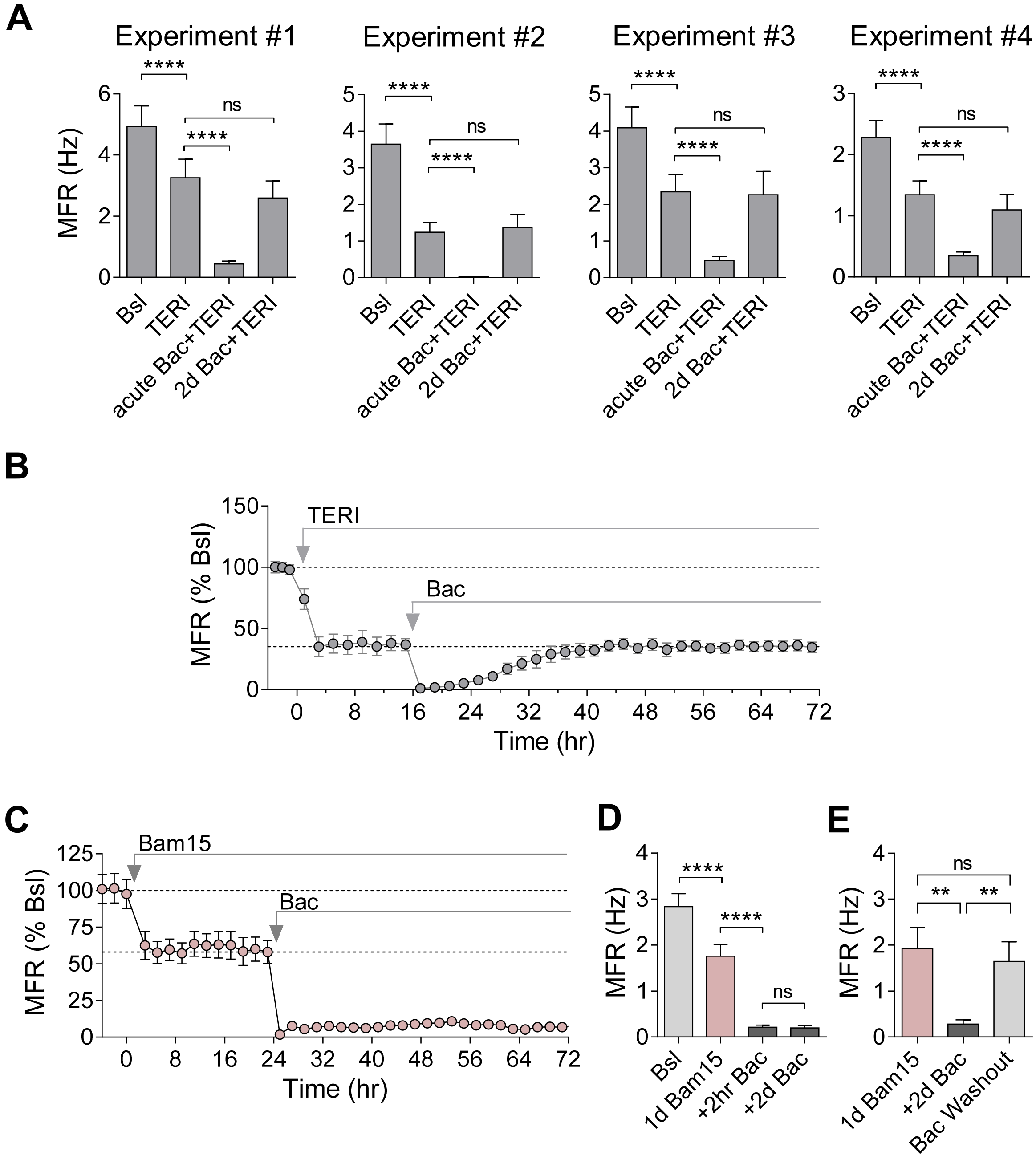

(A) Changes in MFR set-point following DHODH inhibition per experiment. Each tested hippocampal network grown on MEA shows recovery back to the specific lower MFR following TERI (n = 76, 64, 51, 79 channels in experiments #1, 2, 3 and 4, respectively).

(B) An example of experiment with longer MEA recording in the presence of baclofen following TERI shows stable renormalization to a new, lower set-point (63 channels, 1 experiment).

(C-D) Partial mitochondrial uncoupling (1 μM Bam15) stably inhibited MFR to 60% of baseline, while impaired MFR renormalization to baclofen (10 μM) perturbation (n = 107 channels, 3 experiments).

(E) Washout of baclofen following 2 days of application in the presence of 1 μM Bam15 (n = 51 channels, 1 experiment).

One-way ANOVA with Dunnett's multiple comparisons test (A), one-way ANOVA with Tukey's multiple comparisons test (D-E). ****$p < 0.0001$, **$p < 0.01$, ns – non significant ($p > 0.05$). Error bars represent SEM.

Figure S9. Isolation of single units, related to Figure 6

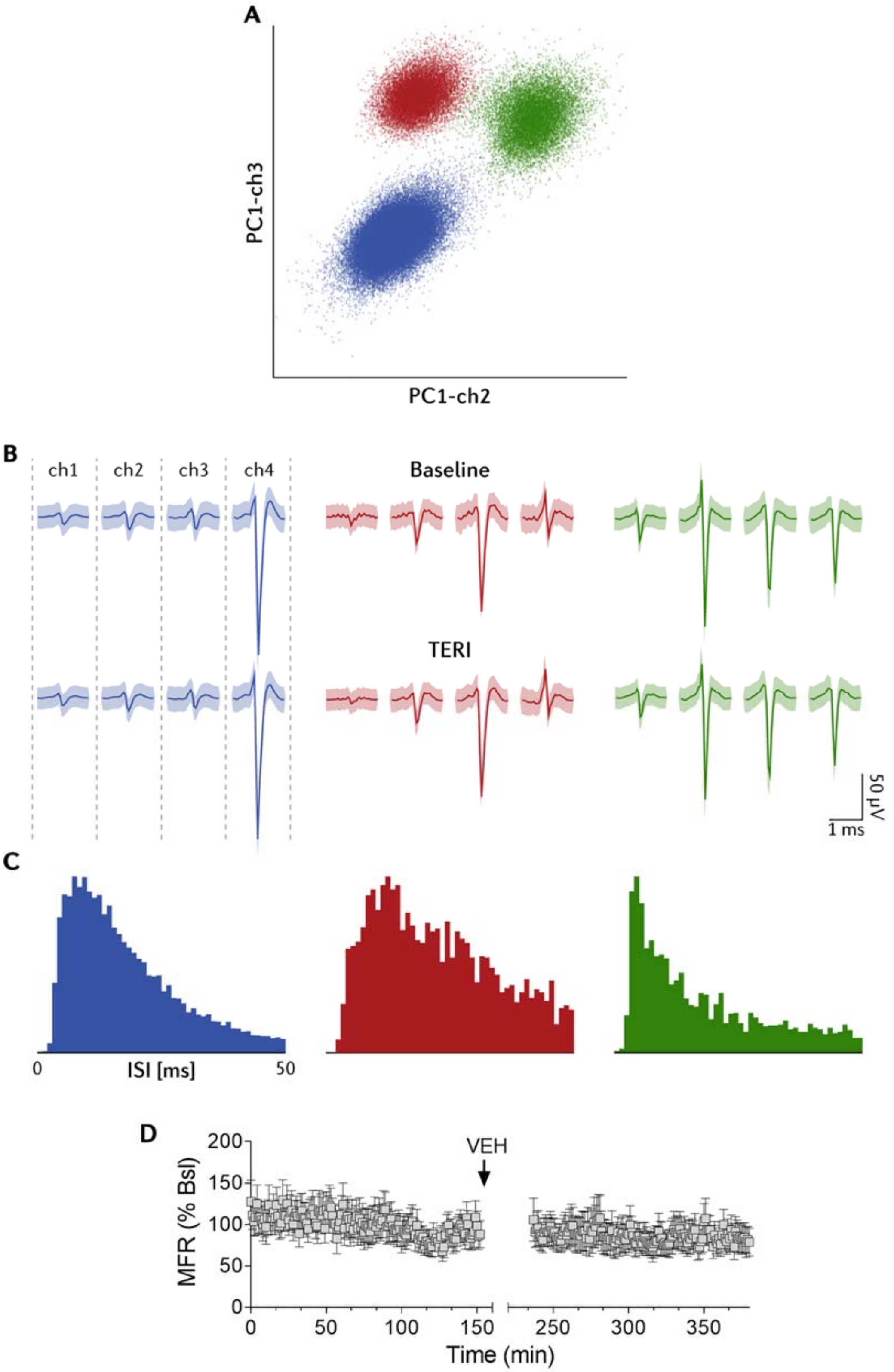

(A) Example of three clusters (color coded) recorded from the same tetrode and projected onto the first principle component of channels 2 and 3. Only well isolated clusters (isolation distance > 20) were included in the analysis.

(B) Mean ± STD waveform traces of the clusters in (A) during baseline recordings (top) and after TERI treatment (bottom).

(C) ISI histogram of the clusters in (A). Only clusters with a well-defined refractory period (less than 1% of inter-spike intervals < 3 ms) were included in the analysis.

(D) Time-course of the effect of VEH (1 µl) i.c.v. infusion on single units MFR in behaving mice (6 mice, 74 single units).

Figure S10. Activity-dependent regulation of mitochondrial and cytosolic Ca^{2+}, related to Figure 7

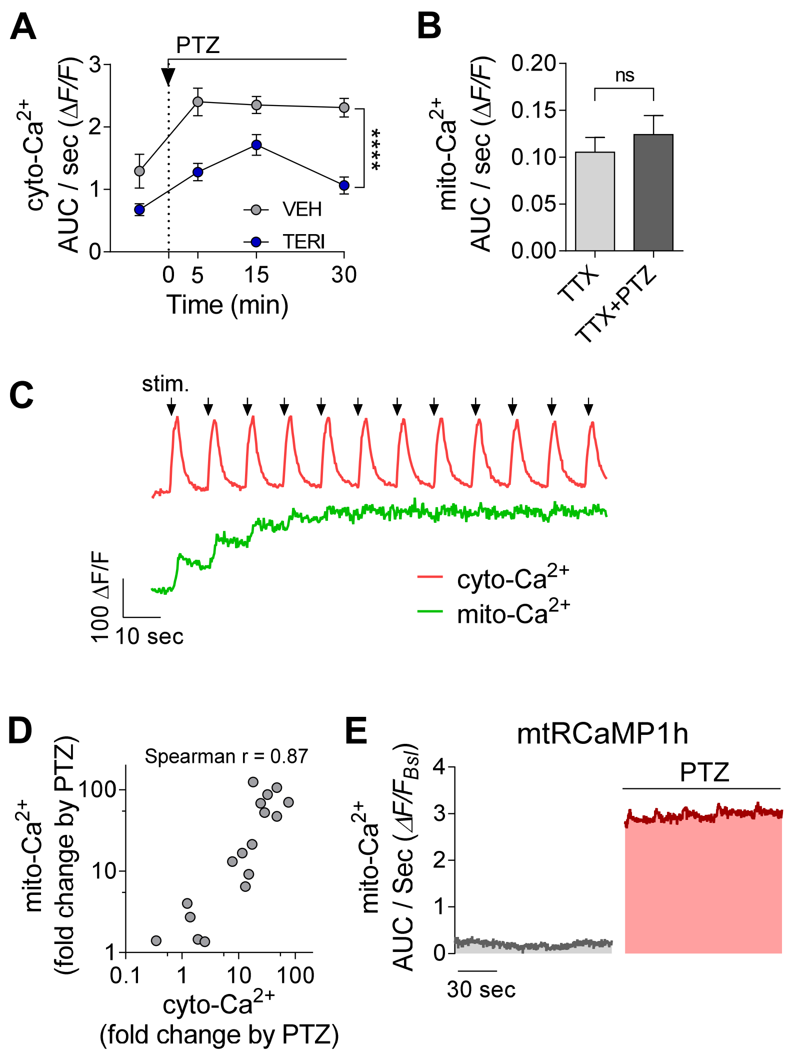

(A) Summary of somatic cyto-Ca^{2+} events at 15 min intervals during 30 min of recording following PTZ application (the same cells as in Figure 7G). Neurons pre-incubated with TERI (50 µM, 1 day; n = 29) showed lower cyto-Ca^{2+} responses compared to those with VEH (n = 18).

(B) PTZ does not affect somatic mito-Ca^{2+} in the presence of TTX (n = 5, p = 0.20).

(C) Effect of electrical stimulation (black arrows, 10 stimuli @ 20 Hz) on somatic cyto-Ca^{2+} (detected by jRGECO1a) and on mito-Ca^{2+} (detected by 2mtGCaMP6m).

(D) Correlation between the effect of PTZ on somatic cyto-Ca^{2+} and mito-Ca^{2+} per neuron ($p<0.0001$, Spearman $r = 0.87$).

(E) Effect of PTZ (10 mM) on somatic mito-Ca^{2+} using mitochondria-targeted RCaMP1h sensor (Kd=1.3 μM).

Two-way ANOWA with *post hoc* Sidak's tests (A) and paired, two-tailed t-test (B) were used for the analysis. ****$p < 0.0001$, ns – non significant ($p > 0.05$). Error bars represent SEM.

Figure S11. Premature death and thermally induced seizures in DS mice related to Figure 8

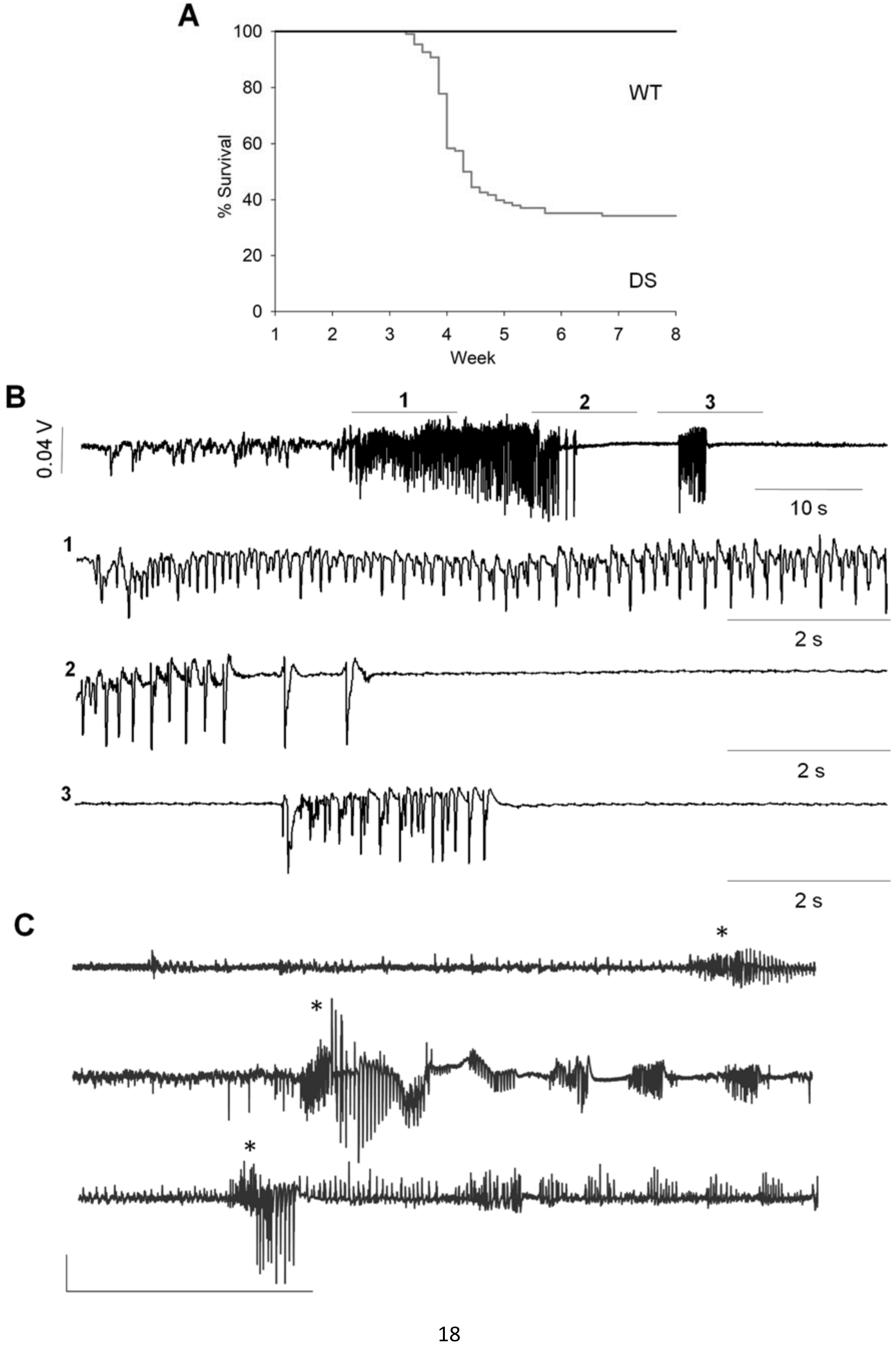

(A) Survival plot of DS (*Scn1a*-A1783V) mice, shown as the percentage of live mice at each postnatal week of age (wild type, n = 101; DS, n = 108).

(B) Intracranial EEG recordings of a thermally induced seizure in DS mice. Mice underwent survival surgery for implantation of EEG electrodes as described (Rubinstein et al., 2015). The top panel depicts a generalized tonic-clonic (GTC) seizure and the lower panels are enlarged view of the indicated segments (1-3).

(C) Representative traces of LFP recorded from CA1 *stratum radiatum* of three different DS model mice, depicting a generalized tonic-clonic (GTC) seizure. Note that the asterisks mark the beginning of each seizure that behaviorally verified to be GTC. Scale bars: 5 mV, 1 minute.

Figure S12. Behavioral scoring matches EEG recordings, related to STAR methods

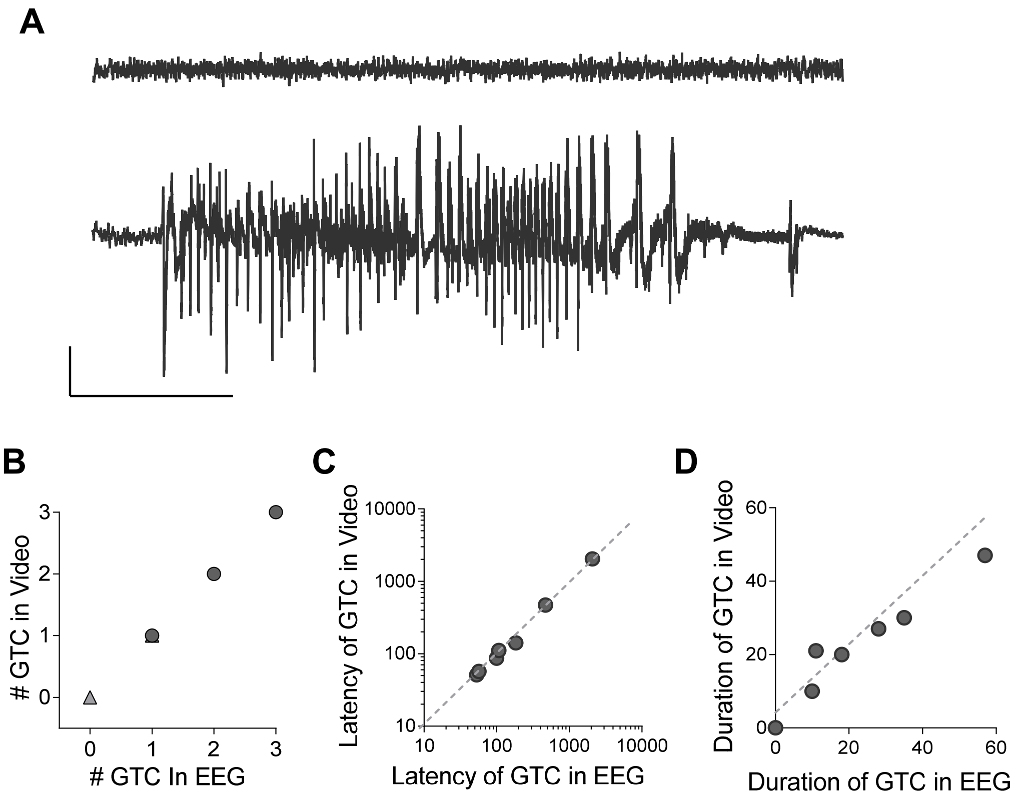

(A) Raw EEG traces showing normal baseline activity (upper trace) and activity from a representative ictus following i.p. PTZ injection (70mg/kg) that was behaviorally verified by video recording to be a generalized tonic-clonic seizure (GTC). Scale bars: 0.5 mV, 5 seconds.

(B-D) Comparison between EEG recordings and behavioral analysis by video monitoring show linear correlation as demonstrated by equal numbers of GTC detected in video and EEG (B), similar latencies to the beginning of each GTC (C, linear regression, $R^2 = 0.99$) and duration of each GTC (D, linear regression, $R^2 = 0.074$).

Table S1. Datasets used for GSM, related to Figure 1 and Figure 8

Dataset	Samples	Source	Platform
Human idiopathic epilepsy	Epileptic patients (24)<br>Non-Epileptic Controls (23)	ArrayExpress<br>E-MTAB-3123	Microarray<br>A-MTAB-540
Kainate rat epilepsy model	Only seizures (5)<br>Sham control (5)	GEO<br>GSE27268	Microarray<br>GPL2882<br>GPL2896
Pilocarpine rat epilepsy	Control (3)<br>Chronic (5)	GEO<br>GSE14763	Microarray<br>GPL2896
Ketogenic diet rat model	Ketogenic diet (6)<br>Regular chow (5)	GEO<br>GSE1155	Microarray<br>GPL341
Dravet mice genetic epilepsy model	P14 [129S6xB6] KO (3)<br>P14 [129S6xB6] WT (3)<br>P24 [129S6xB6] KO no seizures (2)<br>P24 [129S6xB6] KO with seizures (3)<br>P24 [129S6xB6] WT (3)	GEO<br>GSE112627	RNA-Seq<br>Illumina HiSeq 4000

Table 1 notes: The gene-expression data used in this study was obtained from the Gene Expression Omnibus (GEO) https://www.ncbi.nlm.nih.gov/geo/ and from the European bioinformatics institute https://www.ebi.ac.uk/. All samples were taken from the hippocampus, except of the human idiopathic patients where it was derived of cortical tissue. Gene-expression and normalization for each dataset was used in accordance to methods published in the original publications. As the human idiopathic dataset (E-MTAB-3123) didn't have a relevant publication analysis, one was done independently using the R limma toolbox: Significantly expressed genes were selected as genes passing False Discovery Rate Correction (FDR) $P < 0.05$ with a fold change > 1.5. The human orthologous genes were found according to the mouse genome informatics (MGI) Vertebrate Homology database. All datasets were filtered for metabolic genes included in the human metabolic model (Duarte et al., 2007).

Table S2. Identification of seizure-predisposing genes via MTA, related to Figure 1

	Reverse human idiopathic	Reverse chronic kainate	Reverse chronic pilocarpine
**Top 20% reactions**	P= 0.02	P= ns.	P=ns.
**Top 10% reactions**	P=0.004	P=0.04	P=0.003
**Top 5% reactions**	P=0.02	P= 0.009	P= 0.0008

Table S2 note: Enrichment of reverse MTA predictions (i.e. from control to epileptic conditions) within a subset of 38 Knockout-predisposing metabolic genes. In order to validate the usage of MTA in the context of epilepsy, we utilized MTA to predict likely knockouts leading from a healthy metabolic state to an epileptic one. Firstly, we generated a list of seizure predisposing genes by using the MGI phenotype and disease allele query (Search Category: Seizure; MP: 0002064). The resutls of the query were then filtered to include only the reactions maped in the human GSM, thus totalling in 38 metabolic genes which pertubations are known to lead to an increase in seizure activity. In order to define if these reactions were significantly enriched in MTA predictions, we used a hypergeometric statistical test. Reassuringly, a significant enrichment was observed between all three prediction sets and the metabolic MGI seizure predisposing genes, hence establishing a predictive value of MTA in the context of epilepsy.

Table S3. Selected metabolic perturbations predicted to reinstate a healthy metabolic state, related to Figure 1 (See Excel file).

Table S4: MTA predictions converge across the different prediction sets, related to Figure 1

	Chronic Pilocarpine	Human	Ketogenic diet
**Kainate**	63, p=2.38E-11	94, 3.01E-20	59, 2.97E-07
**Human**	55,2.89E-07	x	94, 1.51E-28
**Ketogenic diet**	67, 7.05E-19	94, 1.51E-28	x

Table S4 note: In order to look for genes that are likely to transform a metabolically compromised seizure associated condition towards a healthy or neuroprotective protective one, we have employed MTA to generate four sets of gene predictions. As each data set represents a different pathology, we wondered how preserved are the key metabolic regulators across data sets. A

significant overlap was observed between each MTA prediction set and each anti-epileptic prediction set. This significant overlap indicates that common metabolic processes may regulate divergent metabolic deficiencies.
